# Chemogenomic Profiling of a Plasmodium falciparum Transposon Mutant Library Reveals Shared Effects of Dihydroartemisinin and Bortezomib on Lipid Metabolism and Exported Proteins

**DOI:** 10.1128/spectrum.05014-22

**Published:** 2023-04-17

**Authors:** Camilla Valente Pires, Jenna Oberstaller, Chengqi Wang, Debora Casandra, Min Zhang, Jyotsna Chawla, Swamy Rakesh Adapa, Thomas D. Otto, Michael T. Ferdig, Julian C. Rayner, Rays H. Y. Jiang, John H. Adams

**Affiliations:** a Center for Global Health and Infectious Diseases Research, College of Public Health, University of South Florida, Tampa, Florida, USA; b USF Genomics Program, College of Public Health, University of South Florida, Tampa, Florida, USA; c Department of Molecular Medicine, Morsani College of Medicine, University of South Florida, Tampa, Florida, United States; d Institute of Infection, Immunity and Inflammation, MVLS, University of Glasgow, Glasgow, United Kingdom; e Eck Institute for Global Health, Department of Biological Sciences, University of Notre Dame, Notre Dame, Indiana, USA; f Cambridge Institute for Medical Research, Cambridge Biomedical Campus, University of Cambridge, Cambridge, United Kingdom; National University of Singapore

**Keywords:** *Plasmodium falciparum*, chemogenomics, drug profiles, malaria

## Abstract

The antimalarial activity of the frontline drug artemisinin involves generation of reactive oxygen species (ROS) leading to oxidative damage of parasite proteins. To achieve homeostasis and maintain protein quality control in the overwhelmed parasite, the ubiquitin-proteasome system kicks in. Even though molecular markers for artemisinin resistance like *pfkelch13* have been identified, the intricate network of mechanisms driving resistance remains to be elucidated. Here, we report a forward genetic screening strategy that enables a broader identification of genetic factors responsible for altering sensitivity to dihydroartemisinin (DHA) and a proteasome inhibitor, bortezomib (BTZ). Using a library of isogenic *piggyBac* mutants in P. falciparum, we defined phenotype-genotype associations influencing drug responses and highlighted shared mechanisms between the two processes, which mainly included proteasome-mediated degradation and the lipid metabolism genes. Additional transcriptomic analysis of a DHA/BTZ-sensitive *piggyBac* mutant showed it is possible to find differences between the two response mechanisms on the specific components for regulation of the exportome. Our results provide further insight into the molecular mechanisms of antimalarial drug resistance.

**IMPORTANCE** Malaria control is seriously threatened by the emergence and spread of Plasmodium falciparum resistance to the leading antimalarial, artemisinin. The potent killing activity of artemisinin results from oxidative damage unleashed by free heme activation released by hemoglobin digestion. Although the ubiquitin-proteasome system is considered critical for parasite survival of this toxicity, the diverse genetic changes linked to artemisinin resistance are complex and, so far, have not included the ubiquitin-proteasome system. In this study, we use a systematic forward genetic approach by screening a library of P. falciparum random *piggyBac* mutants to decipher the genetic factors driving malaria parasite responses to the oxidative stress caused by antimalarial drugs. This study compares phenotype-genotype associations influencing dihydroartemisinin responses with the proteasome inhibitor bortezomib to delineate the role of ubiquitin-proteasome system. Our study highlights shared and unique pathways from the complex array of molecular processes critical for P. falciparum survival resulting from the oxidative damage of artemisinin.

## INTRODUCTION

Plasmodium falciparum causes the most severe type of malaria leading to an estimated 241 million malaria cases and 627,000 malaria deaths worldwide in 2020 (1). Of the last remaining frontline antimalarial treatments, artemisinin (ART)-based combination therapies (ACTs) target the symptomatic phase of the disease by killing the parasites during their intraerythrocytic asexual developmental cycle. The exceptional potency of ART against the parasites is well established, but emerging ART resistance (ART-R) (2–6) is leading to increased failures with current treatments. Continued dispersal of ART-R P. falciparum, especially to the regions of high endemicity in Africa (7), could have catastrophic consequences, potentially leading to a global resurgence of malaria (8).

The mechanism of action of artemisinin occurs via volatile reactive oxygen species that are created after activation of its endoperoxide bridge by free heme that is released as a result of hemoglobin digestion by the parasite (9–12). The resultant oxidative damage leads to accumulation of damaged and unfolded proteins that overwhelms the parasite’s stress response coping mechanisms and eventually leads to parasite death (13–15). Based on this model, the proteasome-mediated degradation pathway is hypothesized to be a major component of the stress responses to artemisinin cytotoxicity, and therefore parasite modulation of the proteasome degradation pathway could be a major contributor to ART-R. Numerous studies support this idea by demonstrating a significant link between artemisinin and the proteasome response mechanisms, suggesting the potential use of proteasome inhibitors with artemisinin to reduce the parasite’s ability to tolerate the accumulation of damaged and unfolded proteins (16–18).

A growing body of evidence indicates tolerance of artemisinin by the malaria parasite extends beyond the protein damage response, involving multiple cellular mechanisms that are yet to be unraveled in the complex ART-R phenotype. A number of recent experimental breakthroughs have helped identify some of the other functional changes associated with ART-R, including the K13 resistance mechanism that slows cytostome function, to diminish the available hemoglobin in the food vacuole, thereby slowing release of the free heme (2, 19). Non-K13 mutations implicated in ART-R, such as those of AP-2μ (medium subunit of AP-2 adaptin complex) (20–22), coronin (F-actin-binding protein) (23, 24), falcipain 2 (2, 25–27), and P. falciparum phosphatidylinositol 3-kinase (*Pf*PI3K) (28, 29), might also be expected to affect artemisinin susceptibility by altering hemoglobin uptake and trafficking pathways.

The complexity of the ART-R phenotype necessitates additional approaches to provide a more comprehensive understanding of the processes and pathways that can alter artemisinin sensitivity and resistance in P. falciparum (30, 31). Functional genomics is a promising approach and has been successfully employed to analyze essential processes in *Plasmodium* and other members of the Apicomplexa (32–35). In this study, we phenotypically screened a library of isogenic P. falciparum *piggyBac* (*pB*) mutants by exposing them to the artemisinin derivative dihydroartemisinin (DHA) and the proteasome-inhibitor bortezomib (BTZ). This allowed us to create chemogenomic profiles and define drug-gene interactions, as we have done previously in other contexts (36–38). As rationalized in previous studies, when exposed to a given drug, *pB* mutants with disruptions in genes that are linked to a drug’s mechanism of action will have increased sensitivity and grow poorly compared to mutations in genes not contributing to the parasite’s survival under drug stress. Comparing the response of *pB* mutants to the two drugs allowed us to untangle the role of the ubiquitin-proteasome system from the parasite’s multifaceted responses to artemisinin and define common and distinct gene sets linked to the DHA and BTZ sensitivity. Transcriptome profiling was used to further define the metabolic pathways related to the stress responses associated with these drug responses and identify dysregulated processes in a sensitive mutant. Furthermore, our results linked P. falciparum sensitivity to these compounds with lipid metabolism and protein export, while the regulation of specific factors of the exportome formed a major difference between DHA and BTZ mechanisms of action.

## RESULTS

### Chemogenomic profiling of P. falciparum *piggyBac* mutants.

We identified genes linked to responses to the antimalarial drug DHA, an artemisinin compound, and BTZ, a proteasome inhibitor, using phenotypic screens of a P. falciparum *pB* mutant library. The *pB* mutant library was created by single random insertional mutagenesis and included ~600 genetic mutants (here referred to as the half-K *pB* library) with insertion sites in intergenic, untranslated, or exonic regions (see Fig. S1 in the supplemental material). The half-K *pB* library represents ~11% of the P. falciparum genome and has a broad representation of Gene Ontology (GO) that is like that of the previously defined saturation library (39). Similarly, there is a broad diversity of GO processes related to artemisinin and stress responses (9, 10, 40), such as virulence factors, exported proteins, lipid metabolism, oxidoreductase activity, phosphatase activity, RNA metabolism, vesicular trafficking, and DNA metabolic processes, such as histone binding (see Fig. S1C and Table S1 in the supplemental material).

**FIG 1:**
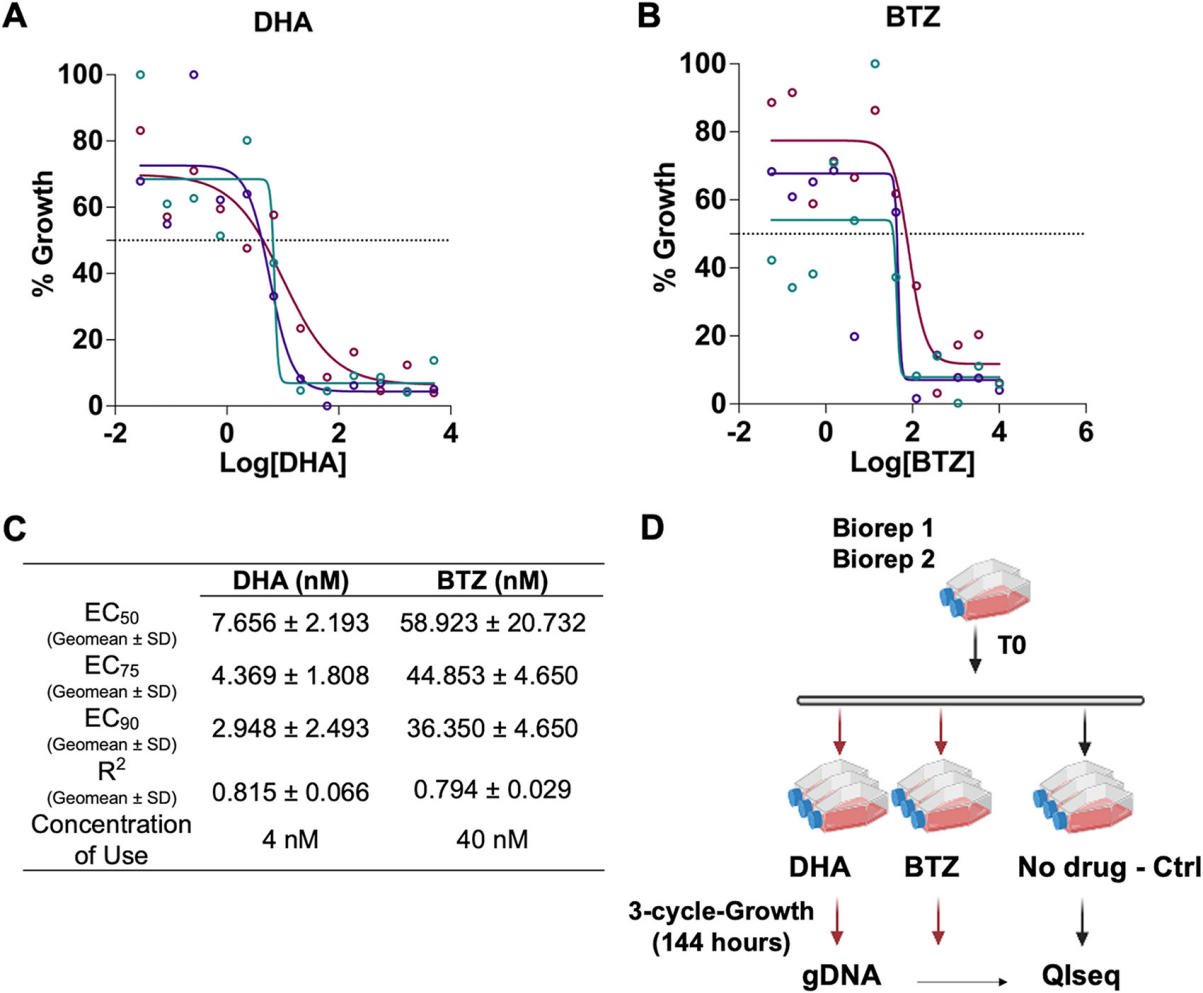
Dose-response curves of (A) dihydroartemisinin (DHA) and (B) bortezomib (BTZ). Standard 72-h drug assays were performed using NF54 (see Materials and Methods); the different colors represent the biological triplicates. (C) Effective concentrations (EC_50_, EC_75_, and EC_90_), *R*^2^ (coefficient of determination) values, and drug concentrations used for the half-K *pB* library drug phenotypic screens; (D) experimental design for DHA and BTZ phenotypic screens. The half-K library (*n* = 553 mutants) was exposed to three cycles (144 h) of continuous drug pressure of sublethal doses of DHA and BTZ. Control flasks were cultured continuously in parallel without drugs. After 144 h of continued drug pressure, the cultures were harvested, and insertion sites were identified by QIseq (quantitative insertion-site sequencing) see (Materials and Methods) (Data Set S1).

The selective pressure for the half-K *pB* library phenotypic screen used sublethal concentrations of DHA (4 nM) and BTZ (40 nM) for three cycles of intracellular growth in parallel with control parasites with no drug exposure (see Materials and Methods) (36, 39, 41) (Fig. 1). Genomic DNA was extracted from each pool before (time zero [T0]) and 1 h after selection (T1), and the relative proportion of mutants present was quantified using quantitative insertion site sequencing (QIseq) (see Materials and Methods) (41) (Fig. 1B; see Data Set S1 in the supplemental material). Mutants with sensitive and tolerant phenotypes for each drug exposure screen were defined by comparing the growth of each mutant exposed to DHA or BTZ with its growth in the absence of drug selection under ideal culture conditions (see Materials and Methods) (Fig. 1; Data Set S2). The reproducibility of the screening protocol and phenotypes were confirmed by comparing the results from this large-scale screen, to a previously performed small-scale screen of the pilot *pB* library (36) and herein with selected individual *pB* mutant clones treated with DHA and BTZ (Fig. S2).

**FIG 2:**
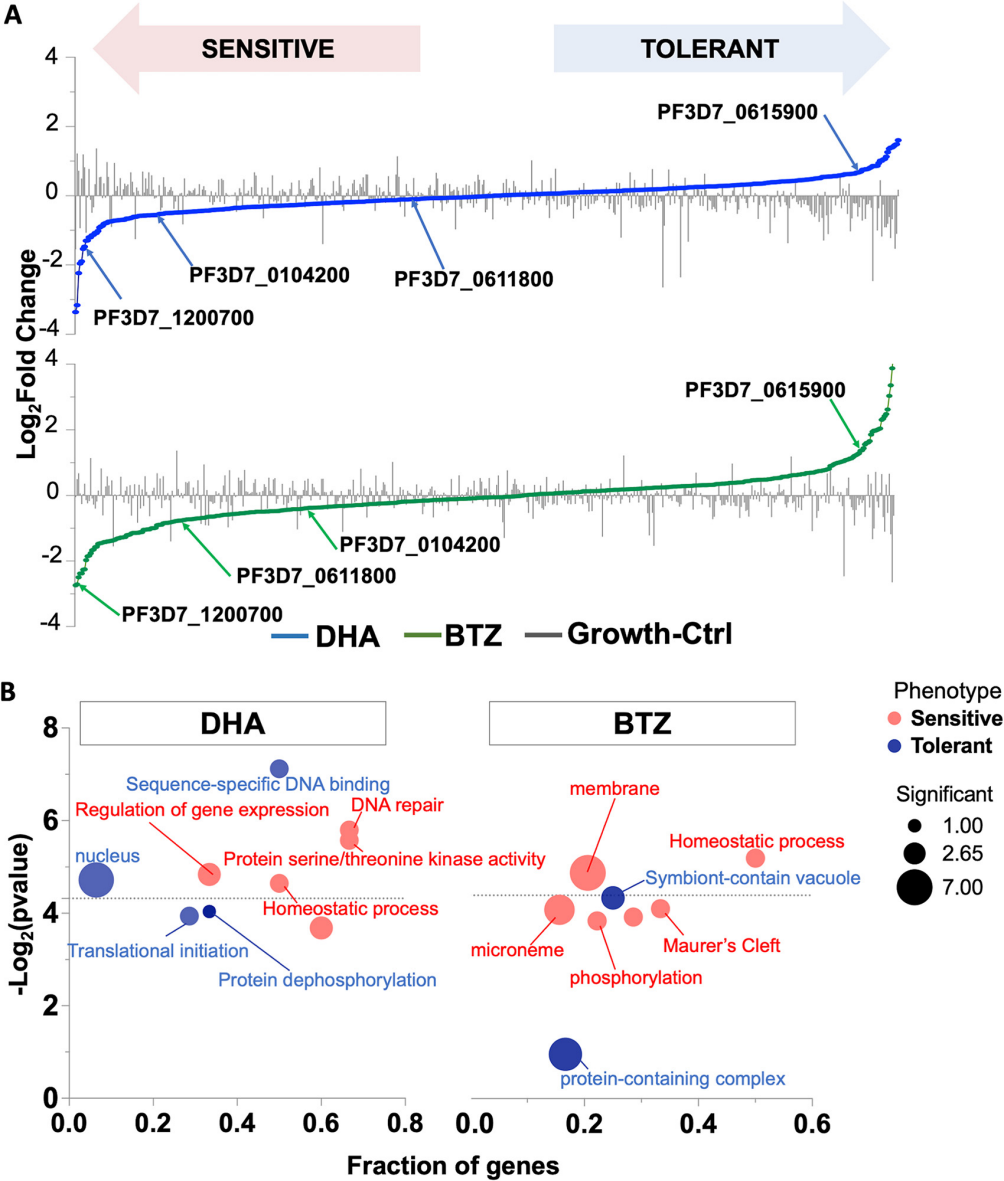
Half-K *pB* library phenotypic screens enable identification of processes underlying the P. falciparum DHA and BTZ responses. (A) Schematic plot showing mutant phenotypes to DHA (blue), BTZ (green), and ideal growth conditions (gray bars) identified in the half-K *pB* library. Aliquots of the library were grown in parallel with DHA, BTZ, and under ideal growth conditions. Mutants were assigned phenotypes by established methods (36). DEseq2 was used for log_2_ fold change calculations and significance assessments (see Materials and Methods). Mutants are ranked from lower (drug-sensitive; lower quartile; *P* < 0.05) to higher (drug tolerant; upper quartile; *P* < 0.05) log_2_ fold change. The mutant’s log_2_ fold changes in ideal growth are superimposed as a bar plot. Internal redundancy within the half-K and pilot clone *pB* libraries and individual drug pressure results showed that phenotypes were reproducible (Fig. S2). Gene IDs and their products are highlighted in the graph: PF3D7_1200700, acyl-CoA synthetases (ACS7); PF3D7_0104200, StAR-related lipid transfer; PF3D7_0611800, conserved *Plasmodium* protein, unknown function; PF3D7_0615900, phosphatase protein. (B) Gene Ontology (GO) enrichment functional analysis of significant cellular components, molecular function, and biological processes of DHA and phenotypes (sensitive or tolerant) along with their corresponding *P* values (above the dotted line; *P* < 0.05). The GO enrichment was performed for each screen, testing GO terms mapped to genes in the category of interest (sensitive, tolerant, and neutral phenotypes) against a background of GO terms mapped to all other genes in the analysis for each screen (see Materials and Methods) (Table S2). Circles represent the GO term. The circle color represents each phenotype (sensitive, red; tolerant, blue), and the circle size represents the number of significant genes annotated to that term. The fraction of genes represents the number of significant genes annotated to a given GO term in each the category being tested for enrichment (category of interest set) divided by the total number of genes annotated to a given GO term included in the analysis for all categories (background set). Significant terms (two-tailed Fisher’s/elim-hybrid test; *P ≤ *0.05) fall above the dotted line.

Mutants with increased sensitivity to both DHA and BTZ were enriched in GO terms described as artemisinin stress responses (9, 10, 40), like homeostasis processes, DNA-repair, phosphorylation, and kinase activity (Fig. 2). Mutants with relative decreased sensitivity to DHA and BTZ (here referred to as tolerant phenotypes) had an expected enrichment in GO processes associated with categories essential for survival under ideal culture conditions (36, 42), such as translation, or a symbiont-containing vacuole (Fig. 2; Table S2).

### Mutations that confer increased sensitivity to DHA and BTZ reveal a shared mechanism of action.

**FIG 3:**
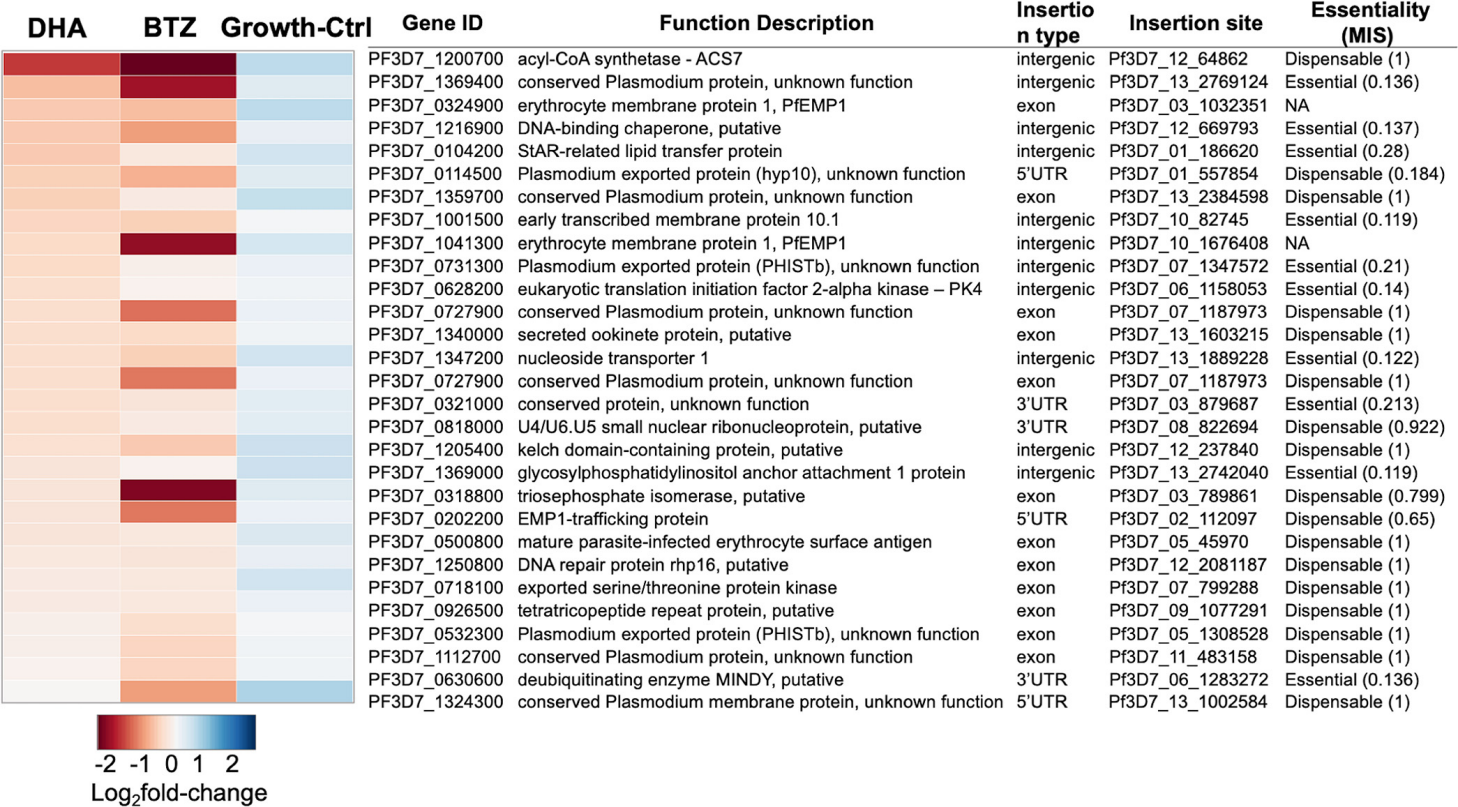
DHA and BTZ overlapped sensitive phenotypes. The heat map shows 29 drug-sensitive *pB* mutants for at least one screen (log_2_ fold change of <0; *P* < 0.05) and with a log_2_ fold change of >0 for the no-drug growth control, meaning growth under ideal conditions is not compromised. The gene IDs, insertion sites, function descriptions, and essentiality status are based on the mutagenesis index score (MIS) from Zhang et al. (39).

A set of mutants common to the DHA- and BTZ-sensitive phenotypes were associated with mutations in proteasome-mediated degradation genes. One such mutant had a mutation in PK4 (eukaryotic translation initiation factor 2-alpha kinase [PF3D7_0628200]), which has been previously implicated in the artemisinin mechanism of action and resistance (16, 17, 37, 39, 40, 43) and well-established artemisinin stress responses (10, 16, 18, 40, 44) (Fig. 3). Mutants with mutations in genes for lipid metabolism (45–50), like the StAR-related lipid transfer (PF3D7_0104200), and fatty acid processes (48, 51–56), such as acyl coenzyme A (acyl-CoA) synthetase-7 (ACS7 [PF3D7_1200700]), also showed an increased sensitivity to DHA and BTZ (Fig. 3). These genes have essential roles in lipid transfer (49, 50) and are involved in the transport of intracellular membranes and membranous structures needed for parasite adaptation and remodeling of the host cell (57, 58). StAR-related lipid transfer protein transports phospholipids (e.g., phosphatidylcholine, phosphatidylinositol, phosphatidylethanolamine, and sphingomyelin) between vesicles associated with endoplasmic reticulum (ER) and mitochondrial membranes (49, 50) (Data Set S3, Tab2). Also, this protein is part of the exportome (50, 59, 60), which is the set of P. falciparum proteins exported into the infected erythrocyte (59, 61–63). The lipid metabolism processes enriched in the sensitive phenotypes (Data Set S3, Tab2) were shown previously to play a role in homeostatic metabolism (55, 56, 64) and to be essential for asexual blood-stage growth (65), including in the artemisinin responses (64, 66). Additionally, mutations in genes for other exported proteins with vacuolar translocation sequence/host targeting motifs (59, 62), such as proteins targeted to the Maurer’s clefts (EMP1-trafficking protein [PTP1; PF3D7_0202200]) and infected red blood cell (RBC) cytosol (PHISTb [PF3D7_0532300], PHISTb/*Pf*G174 [PF3D7_0731300]), also showed increased sensitivity to DHA and BTZ (Fig. 3).

### Differential expression of genes in response to DHA and BTZ.

**FIG 4:**
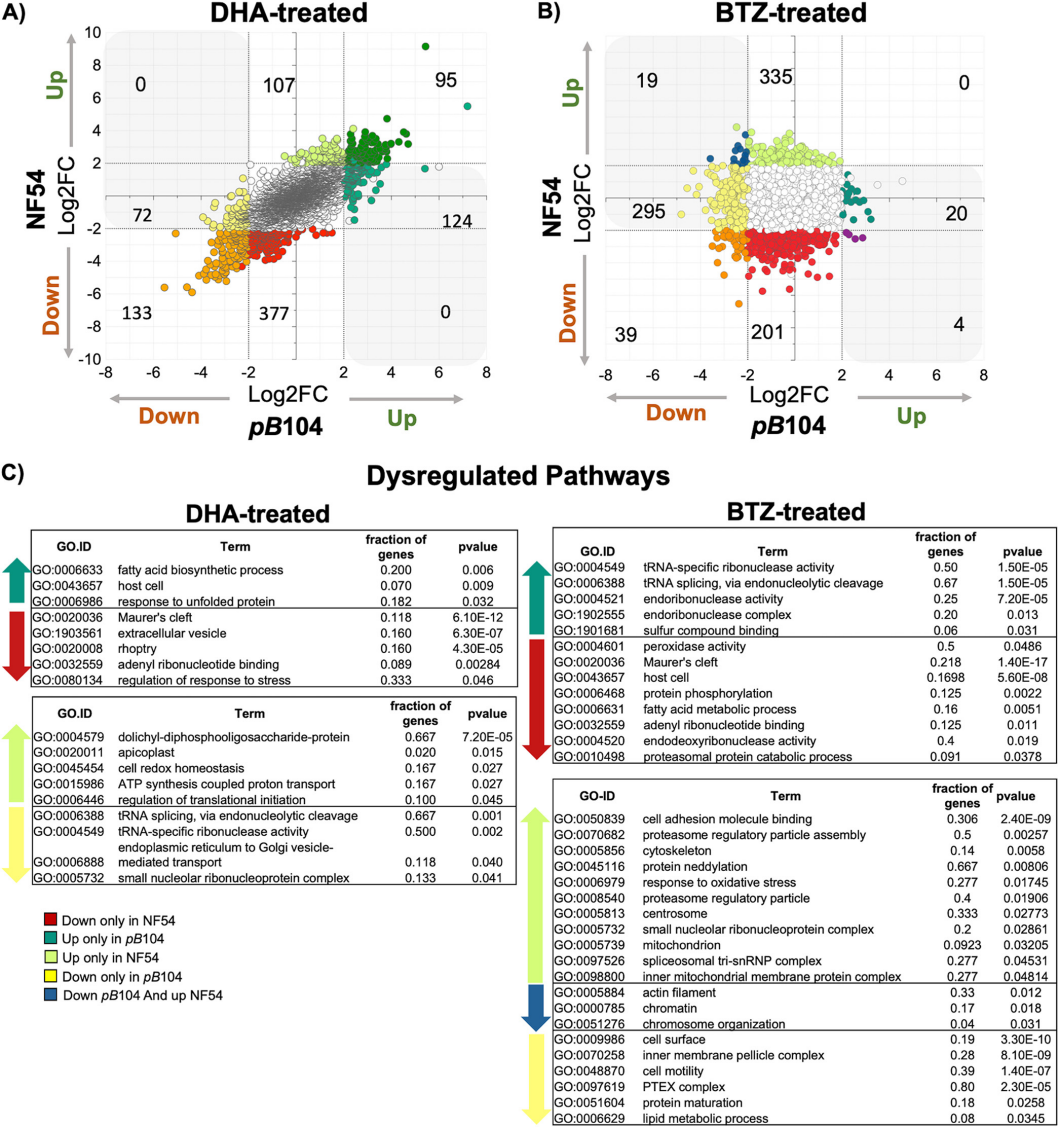
Differential expression of genes reveals parasites with dysregulated expression in the *piggyBac* mutant sensitive to dihydroartemesinin (DHA) and bortezomib (BTZ). Shown is the scatterplot of the log_2_ fold change of individual genes for *pB*104 (*x* axis) compared to NF54 (*y* axis) in (A) DHA and (B) bortezomib. Point color indicates the category of expression genes used to Gene Ontology (GO) enrichment functional analysis (see Materials and Methods) (Data Set S6): white points represent neutral genes (i.e., genes with no significant changes), orange points represent genes downregulated in both parasite lines, red points represent genes downregulated only in NF54, yellow points represent genes downregulated only in *pB*104, light green points represent genes upregulated only in NF54, dark green points represent genes upregulated in both parasite lines, median green points represent genes upregulated only in *pB*104, blue points represent genes downregulated in *pB*104 and upregulated in NF54, and dark reddish-purple points represent genes downregulated in NF54 and upregulated in *pB*104. Shading in gray in the upper left and lower right quadrants signifies genes that are dysregulated—genes with a different direction of regulation comparing *pB*104 and NF54. (C) Relevant GO terms of dysregulated pathways under DHA and BTZ treatment: genes downregulated only in NF54, upregulated only in *pB*104, upregulated only in NF54, downregulated in *pB*104 and upregulated in NF54, and downregulated only in *pB*104. Significant terms were determined by two-tailed Fisher/elim-hybrid test (*P ≤ *0.05) (Data Set S6).

To define a broader network of processes and pathways critical for parasite survival under DHA and BTZ treatment, we performed transcriptome sequencing (RNA-seq) for a *pB* mutant (*pB*104 [PF3D7_0104200]) with altered sensitivity to both DHA and BTZ (Fig. 4; Fig. S2 and S3, Data Sets S4 and S5) (36) and for NF54, the wild-type line. We reasoned that this mutant, having altered sensitivity to both DHA and BTZ, would help unravel the common and distinct responses to DHA and BTZ compared to its isogenic wild-type parent.

The clonal line of *pB*104 carries a *piggyBac* insertion in its intergenic region of StAR-related lipid transfer gene (PF3D7_0104200) (36). Another *pB* mutant in the half-K *pB* library has a *piggyBac* insertion in a similar location to *pB*104 and with similarly altered sensitivity to DHA and BTZ (Fig. S4 and S5) (36). The insertion site of *pB*104 is at the PF3D7_01_186648 position, which is only 28 nucleotides (nt) from the insertion site of the uncloned *pB* mutant in the half-K *pB* library at PF3D7_01_186620. The *pB* insertions are localized between two essential genes, PF3D7_0104200 and PF3D7_0104300 (Fig. S4 and S5) (39, 49, 50). StAR-related lipid transfer gene (PF3D7_0104200) is functionally linked to lipid transfer (50) between organelles and between extracellular and intracellular pathways. The ubiquitin carboxyl-terminal hydrolase-1 (UBP1 [PF3D7_0104300]) is associated with the Kelch13-defined compartment (19). In addition, the DHA- and BTZ-sensitive phenotypes of *pB*104 were validated by individual assays with sublethal drug treatments and the ring-stage assay (RSA) (Fig. S2 and S3).

To characterize the transcriptional changes and the dysregulated pathways of DHA and BTZ responses, we compared the differentially expressed genes for NF54 and *pB*104. For that, we defined gene categories based on the relative significance of gene regulation of NF54 and *pB*104 with and without drug exposure (see Materials and Methods) (Fig. 4; Fig. S6). The comparison of transcriptional profiles of wild-type NF54 and *pB*104 revealed distinct responses to DHA and BTZ in terms of the number of differentially expressed genes. DHA had a significant effect on transcription in both parasite lines—the mutant *pB*104 and wild-type NF54 line—with an upregulation of 95 genes and a downregulation of 133 genes (Fig. 4). In contrast, BTZ had a markedly different effect on the parasite lines: 335 genes were upregulated and 201 genes downregulated exclusively in NF54, whereas 295 genes were downregulated and 20 upregulated exclusively in *pB*104 (Fig. 4).

Next, the GO enrichment analysis showed processes enriched for unfolding protein responses to DHA and BTZ were different (see Materials and Methods) (Fig. 4; Fig. S6 and Data Set S6). In fact, activated DHA initiates a series of reactions to alter protein homeostasis by damaging and unfolding proteins, preventing folding of newly synthesized proteins, and inhibiting the proteasome degradation (16). Specifically here, DHA dysregulated genes related to unfolding protein responses (UPRs) (GO:0006986) (Fig. 4C), by upregulating genes coding for chaperone proteins, such as heat shock proteins HSP86 (PF3D7_0708500) and HSP20 (PF3D7_0816500) only in the *pB*104 mutant (Fig. 4; and Fig. S6); and BTZ dysregulated the proteasomal catabolic process protein (GO:0010498) by downregulating it only in NF54, thereby inhibiting proteasomal activation (Fig. 4C; Data Set S6), and the proteasome assembly regulatory particles (GO:0070682) by upregulating them only in *pB*104 (Fig. 4C; Fig. S6), suggesting that a potential overlap between DHA and BTZ responses occurs in the proteasomal catabolic process but not in proteasome assembly.

The transcriptome signature also revealed notable similarities in the responses to DHA and BTZ that provoke major dysregulation of genes linked to endosymbiotic organelles and lipid metabolism protein-coding genes. Particularly, the effect of DHA seemed to be more consequential on the apicoplast processes, by upregulating the metabolism-related genes (GO:0020011 and GO:0004579, coding for dolichyl-diphosphooligosaccharide proteins), whereas BTZ affected mitochondrial processes (GO:0005739 and the inner mitochondrial membrane protein complex gene GO:0098800), upregulating them. Notedly, these upregulations were observed only in NF54, which were otherwise dysregulated in the sensitive *pB*104 mutant, interpreting as loss function changes (Fig. 4). Importantly, isoprenoid metabolism-related genes and other apicoplast metabolic pathways (67) were downregulated by both DHA and BTZ (Fig. 4; Fig. S5). Specifically, BTZ downregulated 1-deoxy-d-xylulose 5-phosphate synthase (DXS [PF3D7_1337200]) only in NF54, and DHA downregulated 1-deoxy-d-xylulose 5-phosphate reductoisomerase (DXR [PF3D7_1467300]) only in *pB*104. Among lipid metabolism-related processes, DHA upregulated the fatty acid biosynthetic process (GO:0006633) only in *pB*104, while BTZ downregulated fatty acid metabolism processes (GO:0006631) only in NF54. Remarkably, an apicoplast protein-coding gene involved in the fatty acid chain extension step, coding for FabG-3-oxoacyl-[acyl-carrier-protein] reductase (FabG [PF3D7_0922900]) (68), was upregulated by both drugs, with DHA upregulating it only in *pB*104, and BTZ only in NF54 (Fig. 4; Fig. S6).

DHA and BTZ treatment also played roles in the modulation of the pathogenesis-related genes, such as components of the parasite invasion machinery and host cell remodeling/exportome (Fig. 4; Fig. S5 and Data Sets S4, S5, and S6). Maurer’s clefts proteins, MAHRP1 (PF3D7_1370300) (69), the serine threonine protein-coding gene FIKK family (PF3D7_0424700) (70), and heat shock protein HSP70-X (PF3D7_0831700) (71, 72) were downregulated only in NF54 in response to both DHA and BTZ (Fig. S6). Of potential functional relevance to these phenotypes, the knockdown of StAR-related lipid transfer and UBP1 proteins (Fig. S5) seems to reduce the transfer of phospholipids between membrane complexes (45–47, 49, 73) and the transport of hemoglobin to the food vacuole (19), respectively. Consequently, this led to the reduction in the global vesicular transport, perhaps by the downregulation of export trafficking processes, such as extracellular vesicles (GO:1903561) downregulated by DHA only in NF54 (Fig. 4C; Data Set S6) (49, 74). Additionally, while DHA dysregulated in *pB*104 the endoplasmic reticulum (ER) to Golgi vesicle-mediated transport process (GO:0006888), BTZ treatment can play a role in the essential core components of the PTEX complex, by downregulating it only in *pB*104 (Fig. 4C; Fig. S6 and Data Sets S4, S5, and S6). The *Plasmodium* translocon of exported proteins (PTEX) complex is formed during invasion, at the same time as parasitophorous vacuolar membrane biogenesis, when the parasite secretes its components. Disruption of any core PTEX component is detrimental to P. falciparum viability, as well as in the rodent malaria parasite Plasmodium berghei, resulting in dysfunctional protein export and subsequent cell death (75–77).

Genes related to RNA splicing processes were another set of genes dysregulated in the artemisinin-sensitive *pB*104 strain by DHA treatment (Fig. 4), which is consistent with changes observed in ART-R lines (27, 66, 78) and may be functionally related to other changes in RNA metabolism, such as tRNA modification genes reported to be upregulated in K13 mutants exposed to DHA (64). Here, the tRNA splicing machinery was differently regulated in DHA- and BTZ-treated strains, being downregulated only in *pB*104 in response to DHA and upregulated in response to BTZ (Fig. 4C; Fig. S6 and Data Sets S4, S5, and S6).

**FIG 5:**
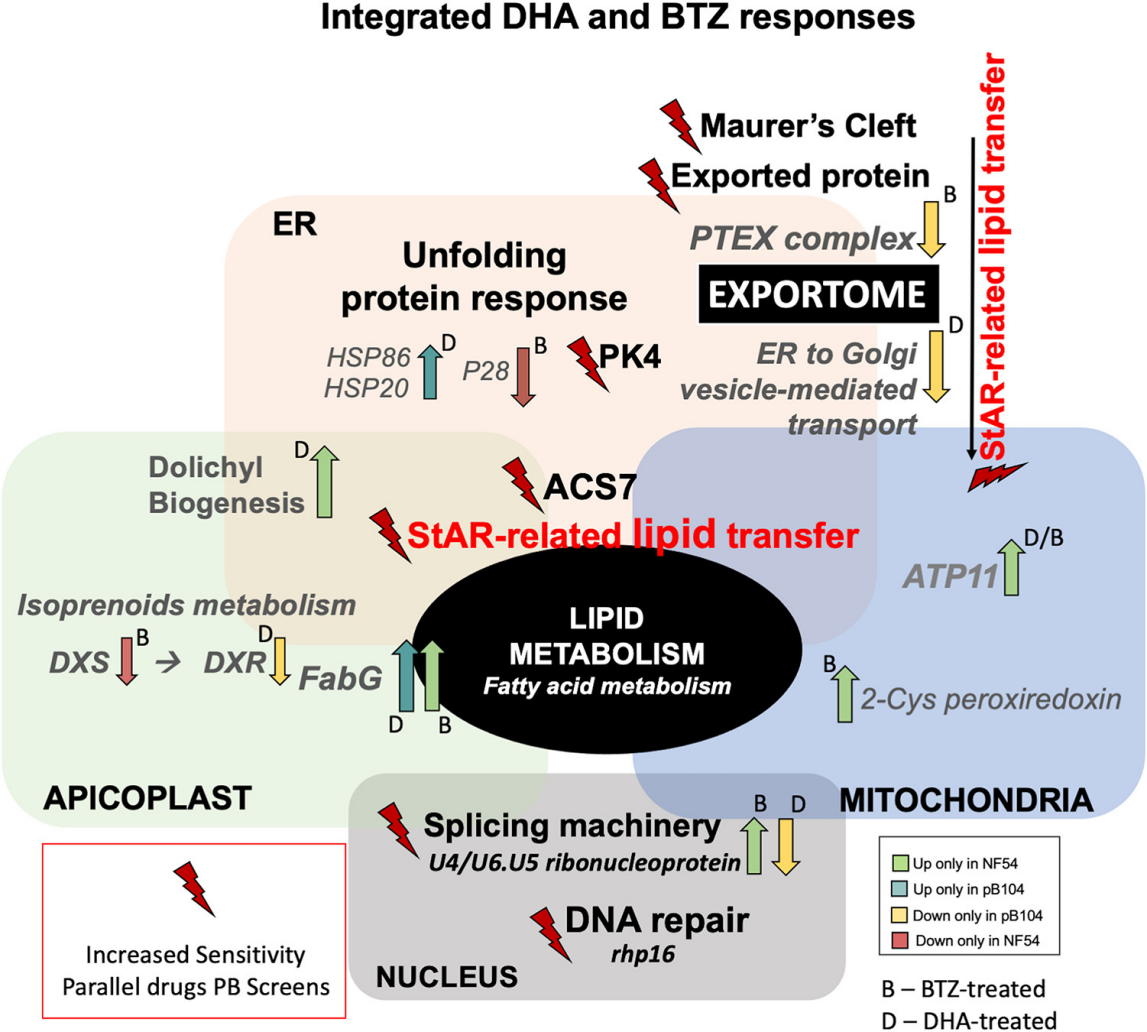
Integrated DHA and BTZ response. All pathways were identified via DHA and BTZ phenotypic genetic screening, and the gene regulation in response to each drug is informed by comparative RNA-seq data where available. DHA- and BTZ-sensitive pathways included the well-known artemisinin mechanism of action-related pathways, such as unfolding protein response (PF3D7_0628200 coding for PK4 [eukaryotic translation initiation factor 2-α kinase]), and DNA repair processes (PF3D7_1250800 coding for DNA repair protein rhp16), as well as gene expression splicing regulation (PF3D7_0818000 coding for the U4/U6.U5 small nuclear ribonucleoprotein). StAR-related lipid transfer (PF2D7_0104200) seems to be the central component of the modulation of lipid metabolism, connecting the organelles’ membranes with the exportome components on membrane lipid trafficking, more specifically for PTEX components related to BTZ-treated regulation (PF3D7_1471100 coding for EXP2 [exported protein 2], PF3D7_1116800 coding for heat shock protein HSP101, PF3D7_1436300 coding for PTEX150—translocon component, PF3D7_1345100 coding for TRX2 [thioredoxin 2], and PF3D7_1105600 coding for PTEX88—translocon component). Proteins involved in fatty acid metabolism, ACS7 (PF3D7_1200700) and FabG (3-oxoacyl-acyl-carrier-protein reductase [PF3D7_0922900]), connect its modulation with apicoplast metabolism, more specifically dolichol biosynthesis and isoprenoid metabolism (PF3D7_1337200 coding for DXS [1-deoxy-d-xylulose 5-phosphate synthetases] and PF3D7_1467300 coding for DXR [1-deoxy-d-xylulose 5-phosphate reductoisomerase]), as well as mitochondrial redox metabolism (PF3D7_0802200 coding for 1-CysPxn [1-cys peroxiredoxin] and PF3D7_1209800 coding for ATP11 [ATP synthase mitochondrial F1 complex assembly factor 1]).

In summary, our data indicate that the synergic effects between DHA and BTZ are in the canonical parasite responses to artemisinin (10, 16, 40, 44, 64, 79–81), such as protein folding machinery and UPR, DNA repair-related processes, and gene splicing regulation. In addition, our phenotype screens demonstrated the importance of the integrative modulation among organelles’ stress responses, including lipid metabolism and the exportome. StAR-related lipid transfer proteins may be responsible for the connection between the exportome and organelles’ metabolism responses, by transferring membranous lipid components (46, 47, 49, 50), and ACS7 connects the organelles’ responses by modulation of fatty acid metabolism (51, 82–84). Furthermore, the dysregulated pathways reveal that part of the difference between DHA and BTZ treatment responses is in the regulation of export trafficking, where expression of the PTEX complex components is specifically altered by BTZ (Fig. 5).

## DISCUSSION

In this study, we investigated the responses of isogenic P. falciparum *piggyBac* mutants to DHA and BTZ to help elucidate the role of the ubiquitin-proteasome system as it relates to the malaria parasite’s response to artemisinin. The chemogenomic profiles from our forward genetic screens combined with transcriptome signatures provided evidence that the different modulation of specific exportome- and organelle-linked lipid metabolism components represent key differences in the P. falciparum response to artemisinin versus ubiquitin-proteasome system. The dysfunction of ACS7 and StAR-related lipid transfer proteins provides a link between the parasite’s organellar stress responses and the transport of phospholipids that alter vesicular trafficking and export-related proteins. Importantly, these findings are consistent with analysis of recent ART-R field isolates wherein exported proteins and lipid metabolism, together with organelles’ metabolism components, are critical to altering sensitivity to artemisinin’s mechanism of action (66, 74, 78, 85).

The results suggest that StAR-related lipid transfer protein connects the exportome to stress response on the organelles. Dysfunction of this gene in the *pB* mutant can explain the increased sensitivity of genes associated with Maurer’s clefts and other exported protein-coding genes to DHA and BTZ treatments (Fig. 3; see Data Set S3 in the supplemental material), which might jeopardize the exportome homeostasis and consequently the essential remodeling of the host erythrocyte. Furthermore, the knockdown of StAR-related lipid protein alters the functionality of the lipid metabolism by altering transport of phospholipids (such as sphingomyelin and ceramide [Data Set S3]) to the organelles’ membranes (49, 50). The lipid metabolism is an integrative part of the parasite’s energy generation, such as the ATP production in the pyruvate and glutamate metabolisms in the mitochondrial functions, which are involved in responses to artemisinin-mediated toxicity (64) and parasite dormancy (55). Thus, the parasite’s response seems to redirect the biosynthetic pathways toward new energy sources, providing acyl-CoAs for the tricarboxylic acid cycle metabolism (64). As acyl-CoA synthases catalyze the elongation steps on the fatty acid metabolism II (FASII) *de novo* pathway and for fatty acid metabolism (48, 51, 54, 55, 82–84, 86), we can speculate that fatty acid metabolism components, including ACS7, aid in this redirecting of energy production pathways. Thus, given that StAR-related lipid transfer is part of the exportome of the P. falciparum (63, 69), and ACS7 was found as to be potential interactor with SBP1 (skeleton-binding protein 1) in the intracellular trafficking (87), our results show a connection between the exportome and lipid metabolism of endosymbiotic organelles in both of the drugs’ mechanisms of action, wherein DHA specifically acts on the apicoplast metabolisms and vesicular trafficking between the ER and Golgi complex, and BTZ acts on the mitochondrial processes and PTEX complex components’ regulation (Fig. 4).

Our findings also confirm a synergy between the effects of artemisinin and proteasome inhibitory compounds on the ubiquitin-proteasome system (16, 40), showing increased sensitivity of protein-coding genes involved in this system, such as the gene coding for PK4 (Fig. 3). DHA induces the phosphorylation of eIF2α by PK4, leading to the global translational attenuation (16, 80). Moreover, the transcriptome profiling also highlights specificity of DHA and BTZ regarding the ubiquitin-proteasome system. DHA leads the inhibition of proteasome components required to dispose of toxic aggregates of damaged proteins (16, 43, 78, 88). We demonstrated that DHA regulates the response to unfolded proteins (HSP86 and HSP20) (Fig. 4 and 5) and that BTZ regulates the proteasome activation by downregulating the proteasomal catabolic processes proteins (Fig. 4) and upregulating the PA28 activator (Fig. 4; see Fig. S6 in the supplemental material).

These observations appear related to how resistant parasites use latency caused by repression of general translation to counter artemisinin-mediated toxicity and upregulate the chaperones and proteasome subunit genes necessary to combat the oxidative assault (78). Whereas tRNA processing is upregulated in ART-R isolates (64, 78), and tRNA modifications modulate the translation efficiency of codon-biased transcripts for critical genes (89), here, the tRNA splicing machinery genes were downregulated only in *pB*104 by DHA treatment and upregulated only in *pB*104 in response to BTZ (Fig. 4). We speculate that modulation of artemisinin mechanism of action includes the splicing and translational mechanisms in regulating gene expression. Alternative splicing that has been described for 4.5% of P. falciparum genes (90) might be a pathway for modulating responses to drug toxicity. Additional investigation is needed to determine what pathways would play a role in splicing regulation to alter ART sensitivity of parasites.

Our genome-wide screening approach provides a platform for identifying the complex array of genetic factors involved in the parasite’s responses to the mechanisms of action to antimalarial drugs. Future studies expanding on our analysis and validating interactive mechanisms of action will further advance our molecular understandings of parasite responses to artemisinin and support the development of efficient drug combination therapies.

## MATERIALS AND METHODS

### Parasite strains and *pB* mutant library culture maintenance.

The wild-type P. falciparum NF54, all *pB* mutant lines, and the half-K *pB* library were cultured in 4% hematocrit (O^+^ erythrocytes from Interstate Blood Bank, Memphis, TN) and 1% Albumax II in RPMI 1640 medium (Invitrogen) supplemented with 50 μg/mL hypoxanthine (Sigma) and 25 mM HEPES (Invitrogen). The culture flasks were grown in an incubator with continuous flow of mixed gas (90% nitrogen, 5% CO_2_, and 5% O_2_).

### Determination of DHA and BTZ sublethal concentrations.

The standard 72-h drug assay was used for the determination of the dose responses of DHA and BTZ. NF54 wild-type line parasites growing in a tightly synchronous culture, using Percoll synchronization for late-stage schizont enrichment (70% to 40% gradient), followed by a treatment with 10% (vol/vol) sorbitol after ~16 h. The ring stages of NF54 line, parental for *pB* mutant lines, at 0.5% parasitemia and 2% hematocrit, were dispensed in plates containing the compounds, serially diluted 1:3 along 12 columns, duplicate per dose, with starting concentrations for DHA of 5 μM and BTZ of 10 μM. The growth response for each compound dilution was obtained by reading the fluorescence generated by DNA intercalating dye SYBR green I (Invitrogen). Plates were analyzed by first reading on the CLARIOstar plate reader for relative fluorescence units (RFU) at the optimal SybrGreen emissions (excitation/emission [Ex/Em] 484-15/528-15). The dose-response curves, with the relevant parameter coefficient of determination (*R*^2^), were plotted and fitted using Prism GraphPad (v.9.3.1). Fifty percent effective concentration (EC_50_), EC_75_, and EC_90_ values were obtained using the same software. Each drug assay was performed in biological triplicate (three independent experiments). The sublethal concentration used this study was ~EC_75_ (geometric mean of all three replicates for each compound): 4 nM DHA and 40 nM for BTZ (Fig. 1A).

### Drug *piggyBac* mutant screens.

Phenotypic drug screens were performed using an ~600 *pB* mutant library (half-K *pB* library) generated from the saturation library (39). A large-scale *piggyBac* screen pipeline of analysis used in Zhang et al. (36) was adapted for the current work. The pipeline consists of three major steps of quality control: (i) protocols were performed using individual *pB* mutant clones (Fig. S2 and S3), (ii) the methods were adapted for pooled screening using the well-characterized pilot library (which includes the individual *pB* mutant clones assayed individually) (36), and (iii) the methods were then scaled up to screen large pools of uncharacterized mutants, representing the half-K *pB* library (Fig. 1C).

Two independent experiments of screening were performed. In each, several aliquots (at least 2 in each round) of the half-K *pB* library were thawed. From the same thawing, the parasites were split into experimental flasks and control flasks. The control flasks were grown under ideal culture conditions to account for the inherent differences in growth rates of the *pB* mutants in the library (36, 39, 41). A time point zero (T0) sample was immediately harvested. Experimental flasks were continuously exposed to drug pressure at sublethal doses: 4 nM DHA and 40 nM for BTZ for three growth cycles (144 h). Use of higher doses, such as EC_50_ or clinical therapeutical doses (700 nM), results in the death of mutants in the pool that greatly diminishes the broader GO representation of the library. As the artemisinin is metabolized rapidly and eliminated within hours (91), old medium was replaced with new medium, and drugs were carefully added every completed 24 h, at due concentrations, during the screening experiment. Parasitemia of all cultures was maintained at 2% during the experiment. The control flasks were cultured continuously in parallel at 37°C without the drug. The parasites were harvested immediately after three growth cycles for genomic DNA extraction and phenotype analysis via QIseq (41).

### Phenotype identification and quantification.

The QIseq methodology was used to identify and quantify *piggyBac* mutants using a modified Illumina sequencing technology and custom library preparation by sequencing from the 5′ and 3′ ends of the *piggyBac* transposon into the flanking genome sequences, and the number of sequence reads per insertion site reflects the relative abundance of each mutant (41) (Data Set S1, Tab2). DEseq2 (41), from R, was used to normalize and calculate fold change between drug treatments (DHA and BTZ) (log_2_ fold change for drug versus control) and no-drug growth control (log_2_ fold change at T1 for the no-drug control versus the T0 no-drug control). The sensitive mutants present a log_2_ fold change of <0 and the tolerant log_2_ fold change of >0, with a *P* value of <0.05 indicating significance. A log_2_ fold change of the growth control of >0 (zero) means growth under ideal conditions is not compromised (36, 41) (Data Set S2). High correlations between biological replicates were observed (Fig. S7).

### Sublethal dose drug exposure and ring survival assay of individual *pB* mutants’ growth.

Phenotype methods for the half-K *pB* library drug screens had been validated previously in small-scale screens (36) and here in growth assays of individual *pB* mutant clones. The wild type (NF54) and *pB* mutant clones associated with StAR-related lipid transfer (*pB*104 [P3D7_0104200]), phosphatase protein (*pB*3 [PF3D7_0615900]), and conserved *Plasmodium* protein of unknown function (*pB*15 [PF3D7_0611800]) were grown with sublethal dose drug exposure and used in the ring survival assay (RSA) (Fig. S3). For the sublethal dose drug exposure (Fig. S3A and B), parasite lines were synchronized in the ring stage by treatment with 5% (vol/vol) sorbitol at 4 h postinvasion (HPI) and diluted down to 0.5% parasitemia at a 4% hematocrit in standard culture medium (described above). Experimental cultures were exposed to 96 h of continuous drug pressure at the sublethal doses: 4 nM DHA and 40 nM BTZ. Controls were cultured continuously in parallel without drug. The medium, containing a fresh dilution of each drug, was changed every day. Giemsa smears were taken every 24 h until 96 h. The parasitemia was estimated by counting, at a ×100 magnification under immersion oil, the number of infected red blood cells (RBCs) containing viable parasites in a total of at least 10,000 RBCs. Each sublethal dose assay was performed in duplicate.

The RSA (Fig. S3C) was performed as previously described (92; https://www.wwarn.org/tools-resources/procedures/ring-stage-survival-assays-rsa-evaluate-vitro-and-ex-vivo-susceptibility). Briefly, parasites were thawed and maintained in culture until reaching the 2 to 4% majority ring stage for sorbitol synchronization. Sorbitol synchronization was again performed 48 h later, depending on parasite development and the stages at the time of sorbitol treatment. After 30 h of culture (majority late schizont stages), cultures were subjected to Percoll synchronization for late-stage schizont enrichment (70% to 40% gradient). The culture was incubated (37°C, mixed gas) for exactly 3 h. Thin smear slides were then taken to evaluate the proportion of ring stages (>0.5%), cultures were incubated with sorbitol, and parasitemia was adjusted to 0.5% at a 2% hematocrit. Tightly synchronous ring-stage parasites at 0 to 3 HPI were then incubated with 700 nM DHA for 6 h, after which the drug-containing medium was washed out, and the parasites’ cultures were allowed to grow into the next invasion cycle for 66 to 72 h. The RSA was performed in triplicate. Giemsa smears were taken, and the parasitemia was estimated by counting, at ×100 magnification under immersion oil, the number of infected red blood cells (RBCs) containing viable parasites in a total of at least 10,000 RBC.

### Comparative RNA-seq between wild-type NF54 and the drug-sensitive *pB*104 mutant parasite line in response to DHA and BTZ.

The RNA-seq experimental design is outlined in Fig. S8. Two independent experiments were performed. Briefly, cultures of wild-type NF54 and sensitive mutant *pB*104 were sorbitol-synchronized 3× to highly synchronous rings, the parasites at time point zero (T0) were harvested and then split equally into experimental (drug exposure) and control (no-drug) flasks. Flasks of each parasite line were then exposed to 96 h of continuous drug pressure at sublethal doses of DHA (4 nM) and BTZ (40 nM), the same concentrations used for the half-K *pB* library screens. Control flasks were cultured continuously in parallel at 37°C without drug. Cultures were maintained under the normal conditions of culture (described above in “Parasite strains and *pB* mutant library culture maintenance”). After 96 h, the RNA was harvested simultaneously from all conditions for RNA-seq as in reference 81. Parasitemia was verified daily by Giemsa smear. RNA-seq was performed in-house on an Illumina NextSeq v.2.5 mid-output 300-cycle, Trueseq reagent kit.

### RNA-seq data analysis.

RNA-seq reads from each sample were aligned to the P. falciparum reference genome (PlasmoDB v.47). A maximum of one mismatch per read was allowed. The mapped reads from HISAT2 (93) and SAMtools (94) were used to assemble known transcripts from the reference, and their abundances were estimated using Feature Counts (95). DEseq2 was using to normalize fold change calculation (drug treated versus control) and significance assessment of differentially expressed genes. The correlations between biological replicates are outlined in Fig. S9. A log_2_ fold change of >2 and adjusted *P* value (*P*adj) of <0.01 classified genes as significantly upregulated for the drug treatment; a log_2_ fold change of less than −2 and *P*adj of <0.01 determined genes as significantly downregulated in response to drug treatment (Data Sets S4 and S5).

### Gene Ontology.

All Gene Ontology (GO) enrichment analyses were performed testing GO terms mapped to genes in the category of interest against a background of GO terms mapped to all other genes in the analysis. The GO term database was created from the latest curated P. falciparum ontology available from PlasmoDB (accessed November 2021), using R package pfGO (v.1.1) (96; https://github.com/oberstal/pfGO), wherein the enrichment was assessed via a weighted Fisher/elim-hybrid *P* value of ≤0.05 (topGO; R package v.2.46.0) (97).

For the GO enrichment analysis of all genes disrupted in the half-K *pB* library (Fig. S1C), the tested categories of interest were “genes disrupted in the half-K *pB* library” versus “genes disrupted in the saturation library” (36) (Table S1).

For GO enrichment analyses of the phenotype of each screen (DHA and BTZ), categories of interest were “sensitives,” “tolerant,” and “neutral” (Fig. 1B; Table S2).

For GO enrichment analysis of RNA-seq transcriptional data, we assigned categories for significant differentially expressed genes based on the relative gene abundance of wild-type NF54 and the *pB*104 mutant. For example, “upregulated-NF54-only” included genes significantly upregulated only in NF54, “downregulated-NF54-only” included genes significantly downregulated only in NF54, “upregulated-*pB*104-only” included genes significantly upregulated only in *pB*104, “downregulated-*pB*104-only” included genes significantly downregulated only in *pB*104, “downregulated-NF54-and-*pB*104” included genes significantly downregulated in both lines, “upregulated-NF54-and-*pB*104” included genes significantly upregulated in both lines, “down-*pB*104-and-up-NF54” included genes significantly downregulated in *pB*104 and upregulated in NF54, and “up-*pB*104-and-down-NF54” included genes significantly upregulated in *pB*104 and downregulated in NF54. All other genes without significance for differential expression were classified as neutral (Fig. 4; Data Set S6).

### Malaria Parasite Metabolic Pathways enrichment analyses.

Complementary to GO-enrichment, pathway enrichment analyses were performed using the Log_2_fold-change ranked sensitive and tolerant genes overlapped in each screen (DHA and BTZ). To access the enrichment, we used a database created from the latest curated P. falciparum Gene Ontology (GO) and Malaria Parasite Metabolic Pathways (MPMP) (http://mpmp.huji.ac.il/) available at the time of analysis, by Fast Gene Set Enrichment Analysis (FGSEA) v.4.2 (98) (Data Set S3).

### Real-time quantitative reverse transcription-PCR gene expression.

Real-time quantitative reverse transcription-PCR (qRT-PCR) was performed using gene-specific primers to both of the nearest genes to the *piggyBac* insertion site in the *pB*104 mutant: genes related to StAR-related lipid transfer (PF3D7_0104200) and UBP1 (PF3D7_0104300) (Fig. S5). The PF3D7_071770 (seryl-tRNA synthetase) gene was used as an endogenous control. To validate the RNA-seq gene expression, total RNA of NF54 and *pB*104 lines was first extracted from ring-synchronized parasite cultures at time point zero (T0) and time point 96 h (T96), using the TRIzol-chloroform method as described previously. First-strand cDNA synthesis was carried out with 500 ng of each sample using oligo(dT)-primed reverse transcription with SuperScript II reverse transcriptase (Invitrogen) according to the manufacturer's instructions. The cDNA was then used as a template for real-time quantitative PCR (qPCR) using Luna Universal qPCR master mix (New England BioLab catalog no. M3003) according to the manufacturer's instructions. The threshold cycle (ΔΔ*C_T_*) method was used to analyze the relative changes in expression level for each gene, using T0 as a calibrator sample (99). All qPCRs were performed in triplicates. The following primers were used for qPCR: (i) for the StAR-related lipid transfer gene (PF3D7_0104200), forward primer AGAAGAATCTCTTGAGACTGCTGCT and reverse primer CTCCCTTCTGCTTCTGCTTGAGC; (ii) for the UBP1 gene (PF3D7_0104300), forward primer TGCCTTAAAGTGGAATGAAATTATATCCC and reverse primer GTCCTGATGATGCTGATATCCCACC; and (iii) for the seryl-tRNA synthetase gene (PF3D7_0717700), forward primer AAGTAGCAGGTCATCGTGGTT and reverse primer TTCGGCACATTCTTCCATAA.

### Whole-genome sequence analysis.

Whole-genome sequence analysis was performed to confirm the *piggyBac* insertion location and to search for eventual additional mutation, comparing NF54 with *pB*104 genomes. Genomic DNA of NF54 and *pB*104 were used for whole-genome sequencing, using the Illumina NextSeq550, Mid Out-put kit. Raw reads were mapped to the Plasmodium falciparum 3D7 reference genome (PlasmoDB v.58) using mapped reads from HISAT2 (93). The sequence alignment data were then postprocessed using SAMtools (94). The Integrative Genomics Viewer (IGV) (100) was used to visualize reads mapped at the *piggyBac* insertion site (Fig. S4B). Bedtools (v.2.28.0) (101) was used to comparative analysis to evaluate the presence of eventual additional mutations, such as SNPs and indels.

### Data availability.

Raw QIseq data sets generated for this study were deposited to the European Nucleotide Archive under study accession code ERP114305 and are presented as follows in the format sample ID primer 3′, primer 5′, accession no.: T0_NoDrug_1_1_3, T0_NoDrug_1_1_5, ERS3788434; T0_NoDrug_1_2_3, T0_NoDrug_1_2_5, ERS3788441; T0_NoDrug_2_1_3, T0_NoDrug_2_1_5, ERS3788447; T0_NoDrug_2_2_3, T0_NoDrug_2_2_5, ERS3788457; T2_NoDrug_1_1_3, T2_NoDrug_1_1_5, ERS3788436; T2_NoDrug_1_2_3, T2_NoDrug_1_2_5, ERS3788442; T2_NoDrug_2_1_3, T2_NoDrug_2_1_5, ERS3788449; T2_NoDrug_2_2_3, T2_NoDrug_2_2_5, ERS3788458; T4_NoDrug_1_1_3, T4_NoDrug_1_1_5, ERS3788438; T4_NoDrug_1_2_3, T4_NoDrug_1_2_5, ERS3788444; T4_NoDrug_2_1_3, T4_NoDrug_2_1_5, ERS3788453; T4_NoDrug_2_2_3, T4_NoDrug_2_2_5, ERS3788460; T2_BTZ_IC25_1_1_3, T2_BTZ_IC25_1_1_5, ERS3788437; T2_BTZ_IC25_1_2_3, T2_BTZ_IC25_1_2_5, ERS3788446; T2_DHA_IC25_2_1_3, T2_DHA_IC25_2_1_5, ERS3788450; and T2_DHA_IC25_2_2_3, T2_DHA_IC25_2_2_5, ERS3788461.

Original QIseq read mapping data are provide in Data Set S1, Tab 2.

RNA-seq data generated for this study have been deposited in the NCBI Gene Expression Omnibus (GEO) database under accession no. GSE188542. Processed RNA-seq data are provided in Data Sets S4 and S5.

Whole-genome sequence data generated for this study have been deposited in the NCBI Sequence Read Archive (SRA) database under accession no. SAMN31408180 for wild-type NF54 and SAMN31408181 for *pB*104.
