# Supplementary material for: Chemogenomic Profiling of a Plasmodium falciparum Transposon Mutant Library Reveals Shared Effects of Dihydroartemisinin and Bortezomib on Lipid Metabolism and Exported Proteins
Source: Microbiol Spectr. 2023 Apr 17;11(3):e05014-22. doi: 10.1128/spectrum.05014-22 (PMC10269874; doi:10.1128/spectrum.05014-22)
Supplement: Supplemental file 1 — Fig. S1 to S9. Download spectrum.05014-22-s0001.pdf, PDF file, 4.7 MB [file spectrum.05014-22-s0001.pdf]

Figure S1

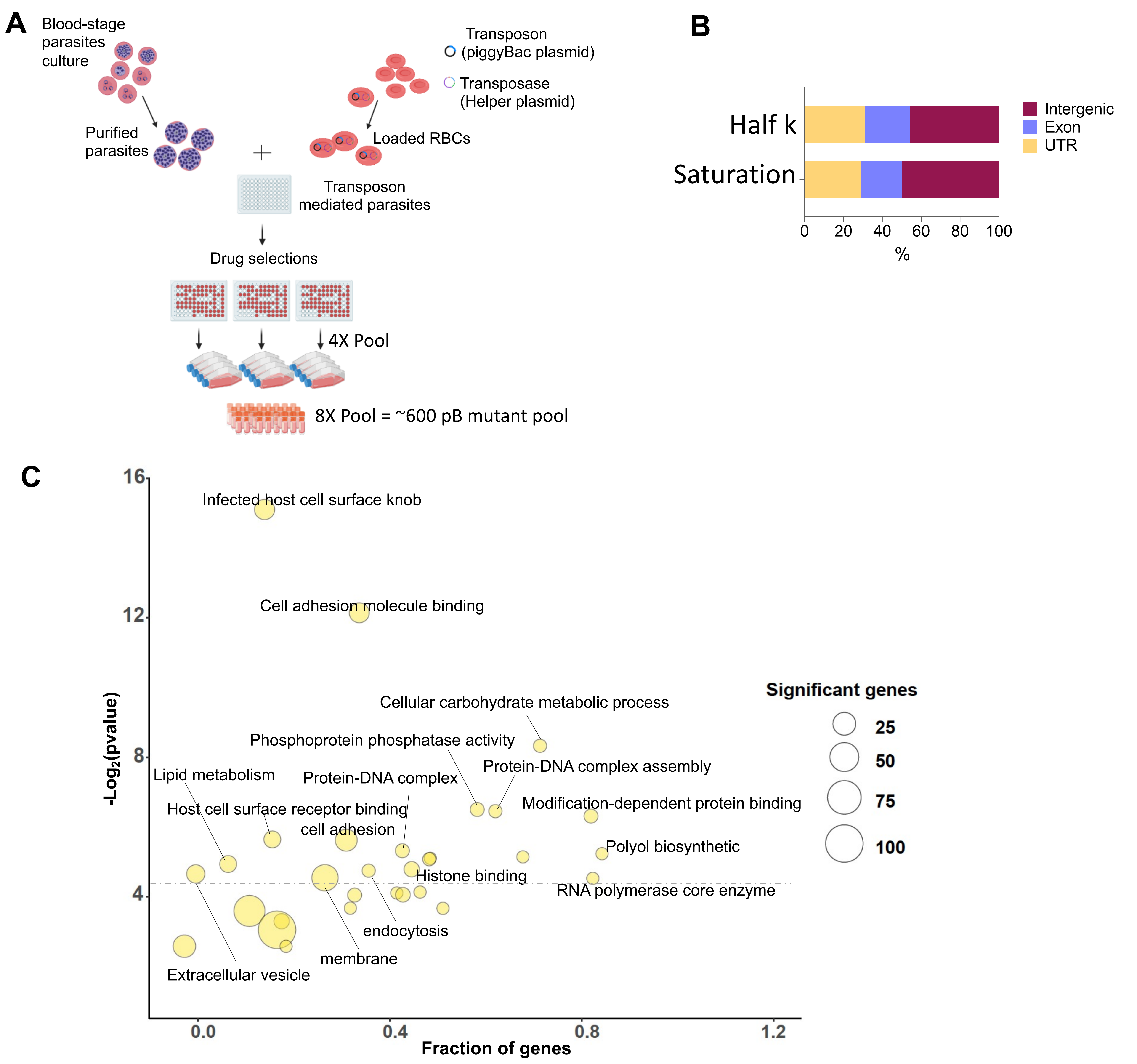

Figure S1. A) Experiment approach for random genome saturation mutagenesis optimized to generate mutants with a single *piggyBac* insertion (Zhang *et al* 2018). B) Proportion of each type of *piggyBac* insertion in the half k *pB* library and saturation library from Zhang *et al* 2018. C). Gene Ontology (GO) enrichment functional analysis of significant cellular components, molecular function, and biological processes, for the half K *pB* library, along with their corresponding pvalues (above dotted line pvalue<0.05). The GO enrichment was performed testing GO-terms mapped to genes in the category of interest (all genes disrupt in the half k *pB* library, and all genes disrupt in the saturation library, Zhang et al 2018) against a background of GO-terms mapped to all other genes in the analysis (see Method and Table S1).

Figure S2

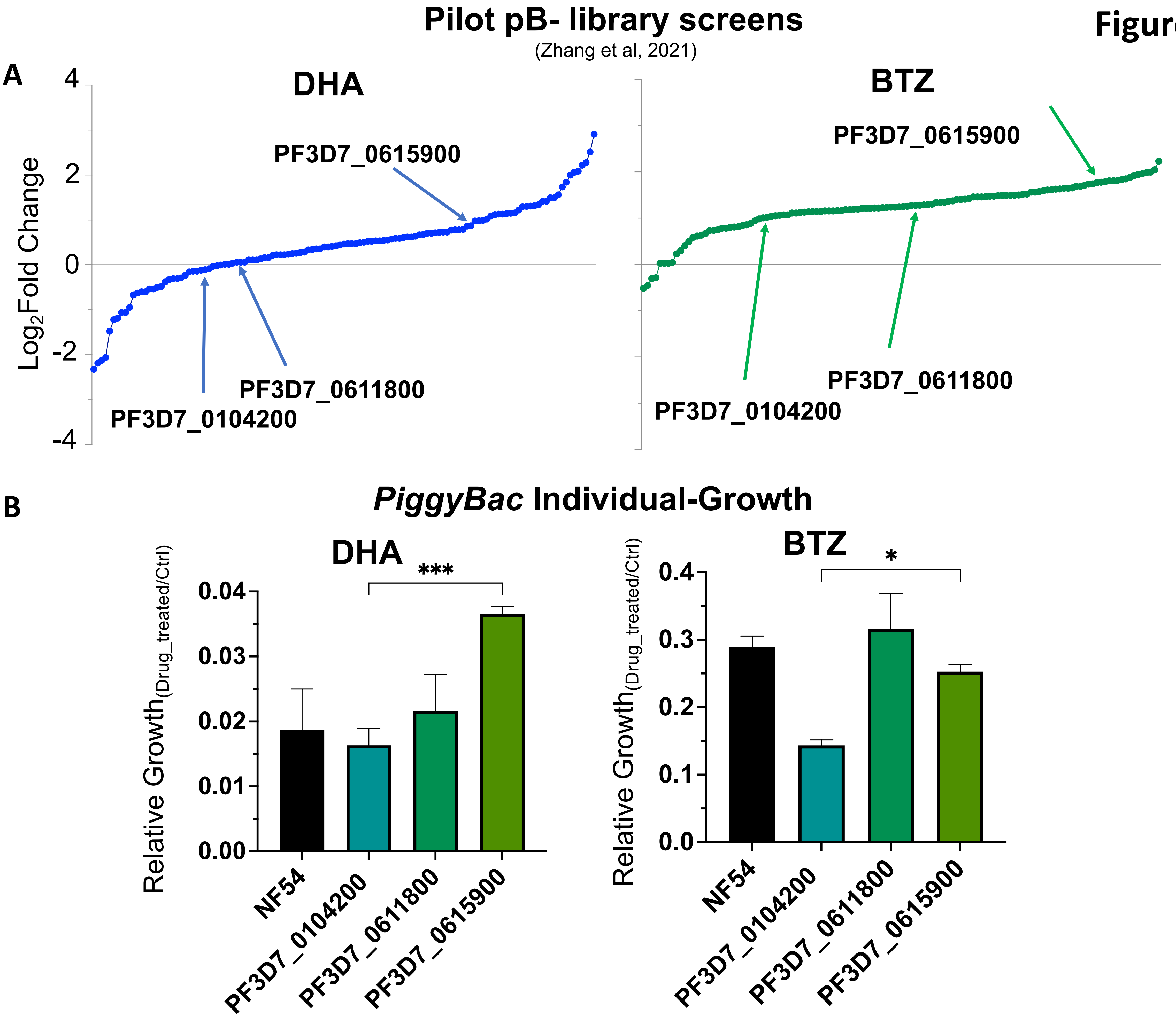

C

GeneID	Function Description	MIS	PB mutant ID	insertion site	Insertion location
**PF3D7_0104200**	StAR-related lipid transfer protein	0.28	pB104*	PfNF54_01_m1::186648	5-UTR
				PfNF54_01_m1::186620	5-UTR
**PF3D7_0611800**	conserved Plasmodium protein, unknown function	1	pB15*	PfNF54_06_m1::498591	Exon
				PfNF54_06_m1::507016	Exon
**PF3D7_0615900**	protein phosphatase, putative	1	pB3*	PfNF54_06_m1::667365	Exon
				PfNF54_06_m1::664011	Exon

Figure S2. DHA and BTZ Phenotype of pooled and individual *piggyBac* mutant growth. A) Pilot *pB*-library (performed in Zhang *et al* 2021). The Log2Foldchange drug/control were ranked from sensitive values (Log2Foldchange <0) to tolerant phenotypes (Log_2foldchange>0). B). Individual growth for DHA and BTZ, upon sublethal dose of drug pressure (see Method). Pool library screens (Pilot and half K) and individual growth were performed for 3-cycles-growth (144 hours) continue drug pressured. Statistical significance was determined by One way ANOVA tests follows by Dunnet's T3 multiple comparison test (*p < 0.05; ***two-tailed Wilcoxon p < 0.001); error bars represent the standard deviation. C). Three *pB* mutated genes, their characteristics and descriptions and their *piggyBac* insertion site.

Figure S3

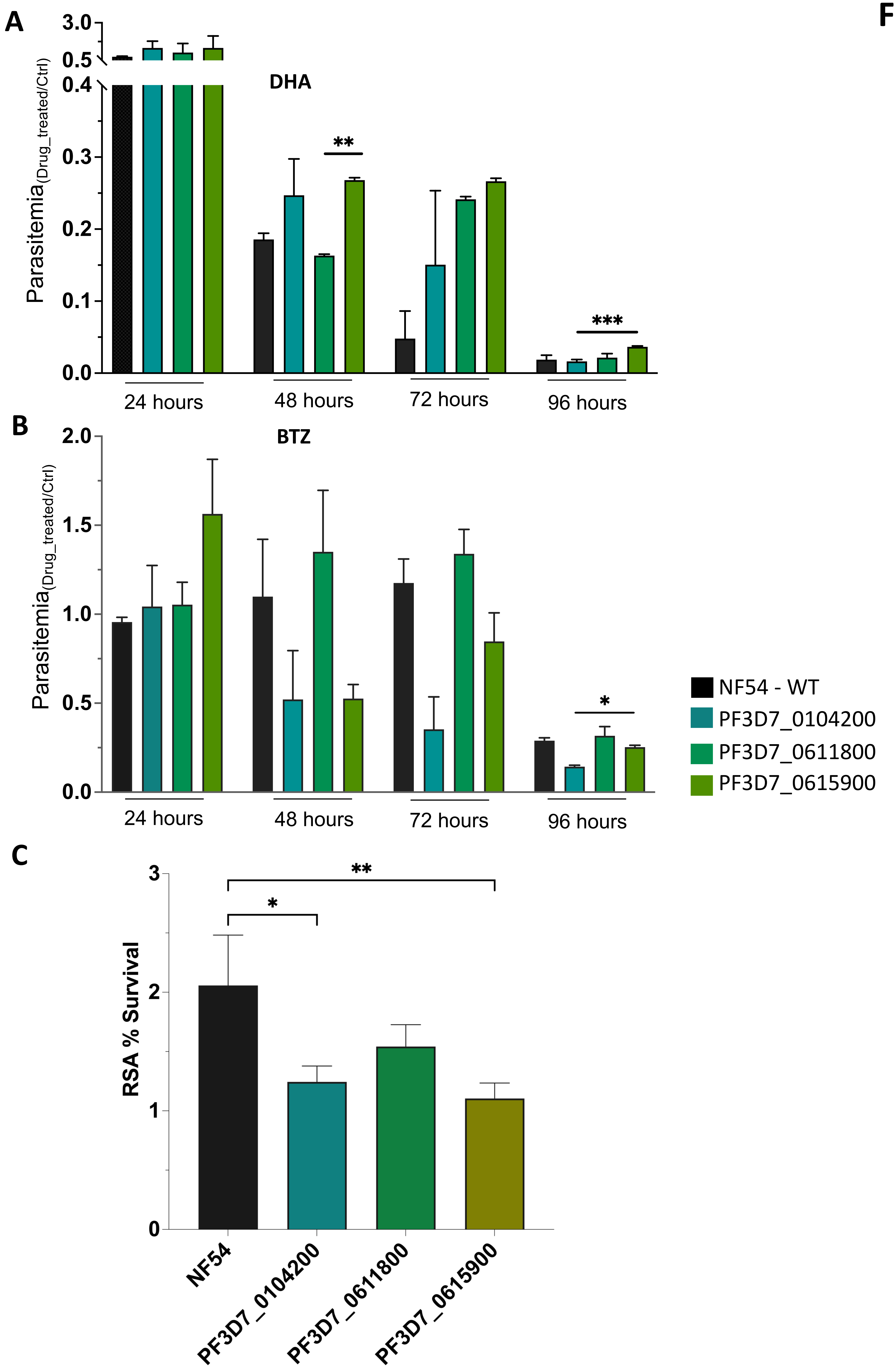

Figure S3. Time course of proportion of parasites (parasitemia of drug_treated divided by no drug control) pressured with sublethal dose with **A)** DHA and **B)** BTZ (see Method). C). Ring Survival Assay (RSA). Statistical significance was determined by One way ANOVA tests follows by Dunnet's T3 multiple comparison. (*p < 0.05; ** pvalue <0.01*** p < 0.001); error bars represent the standard deviation.

Figure S4

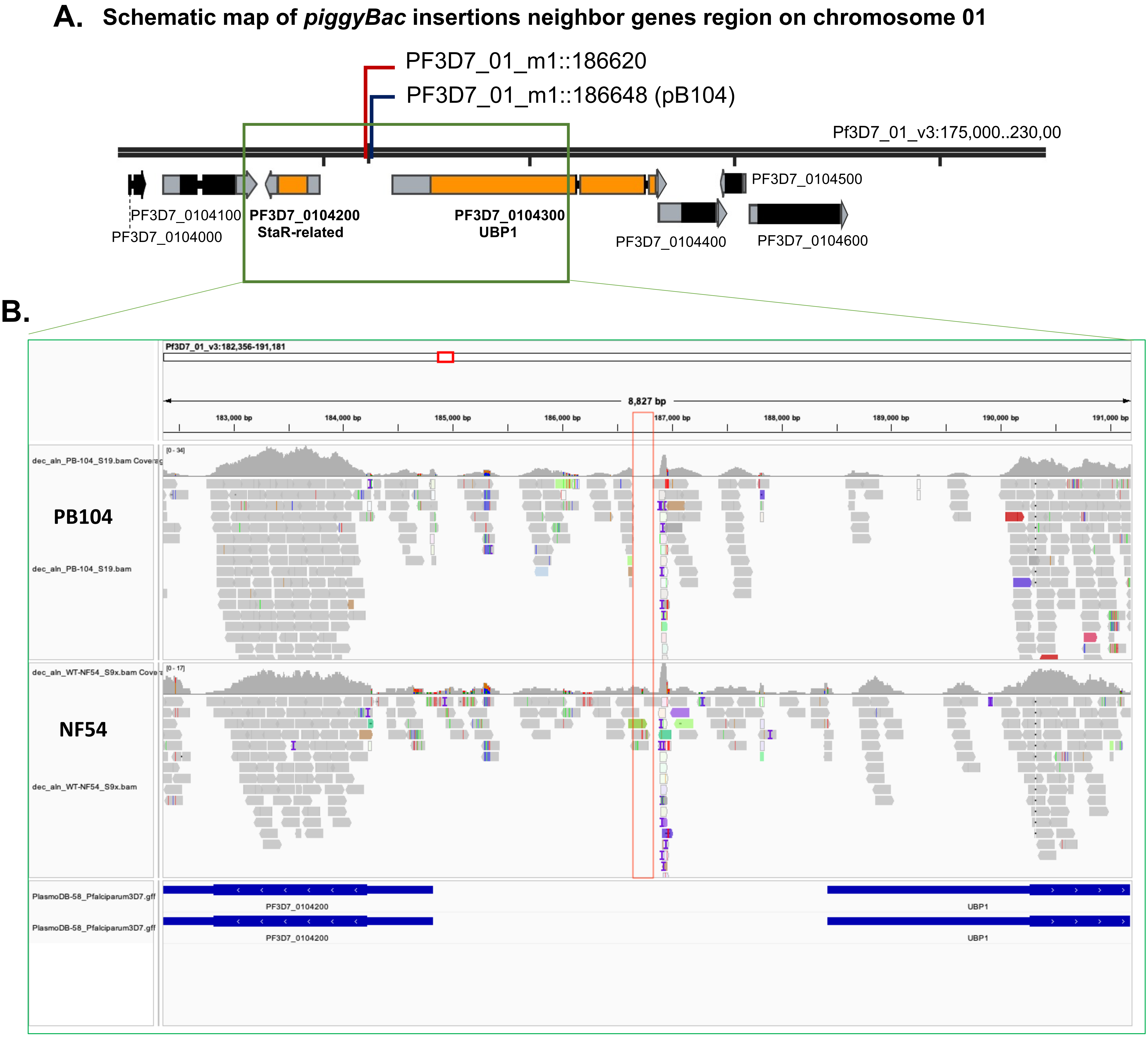

A) Schematic segment of chromosome 01 of *Plasmodium falciparum* 3D7 show two *piggyBac* insertions PF3D7_01_m1::186620 (Half K *pB*-library) and PF3D7_01_m1::186648 (*pB*104 – Pilot-library) placed in a regulatory region on 5'-end of two essential genes StaR-related lipid transport (PF3D7_0104200) and PF3D7_0104300 UBP1 (ubiquitin carboxyl-terminal hydrolase 1). Green box shows the region highlights in B). B). Integrative Genomics Viewer (IGV) screenshot showing the alignment of whole genome sequence reads mapping in 3D7 *P. falciparum* genome (see method) of *pB*104 and NF54 at the Chromosome 1 region between 182,356 to 191,181, highlighting the absence of reads at *piggyBac* insertion site in *pB*104 genome and presence of reads in NF54 genome (red box).

Figure S5

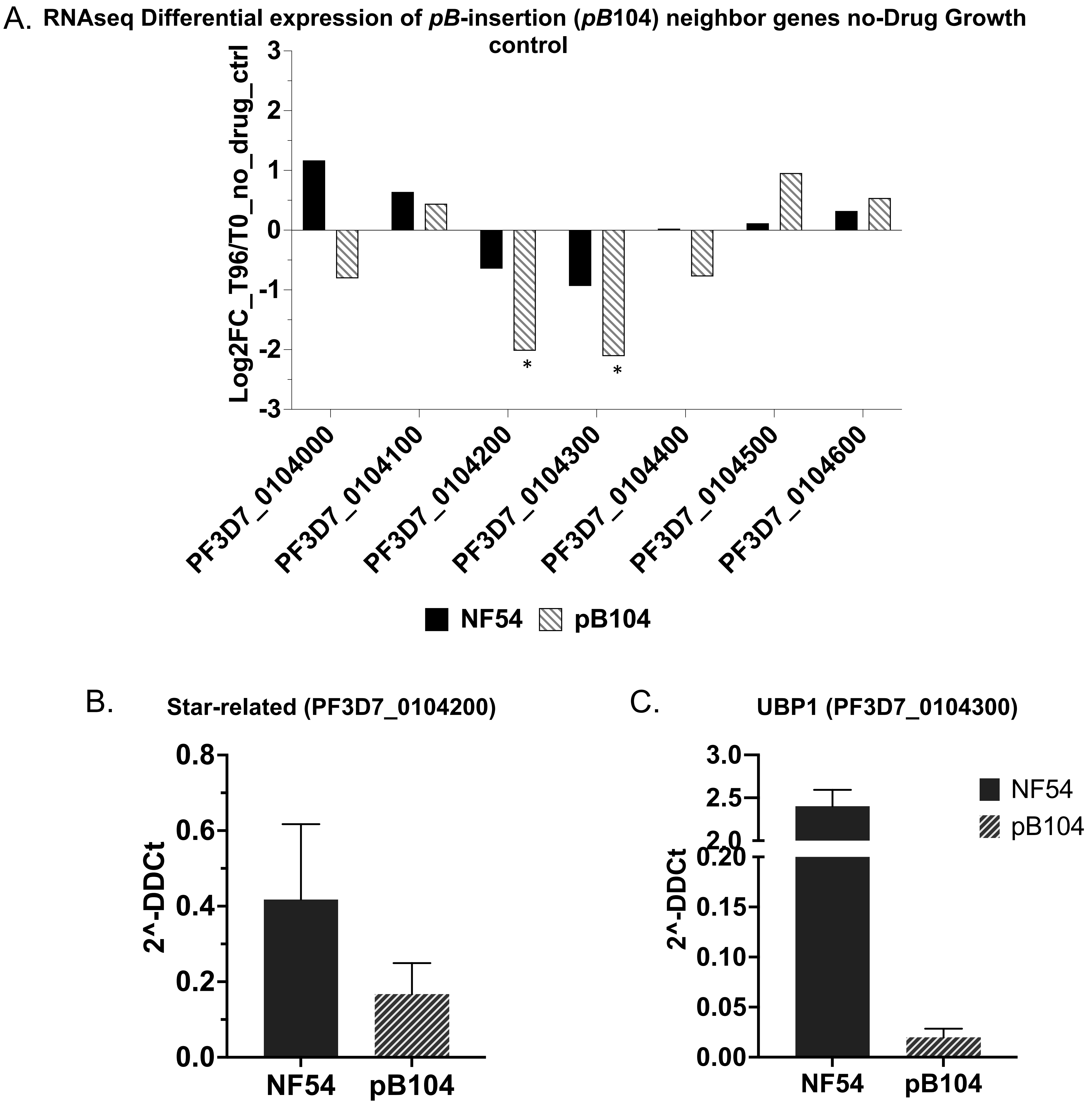

Figure S5. A) Differential expression of *piggyBac* in pB104 insertion neighbor genes obtained by RNAseq data analysis. Deseq2 was used to normalize and calculated the foldchange between T96 and T0 of no-drugs growth-controls. StaR-related lipid transport (PF3D7_0104200) and ubiquitin carboxyl-terminal hydrolase 1 (UBP1, PF3D7_0104300) are significant downregulated (*Log_2foldchange<-2 and padj<0.01) in *pB*104, but not in NF54. The entire data are provided at Data set S4 and S5. B) StaR-related lipid transport (PF3D7_0104200) and C) UBP1 (PF3D7_0104300) gene expression (RT-qPCR) in NF54 and pB104. 2-ΔΔCt is taken as the fold-change of the relative to time point 0, (T0) gene expression; error bars represent the standard deviation (see Method).

Figure S6

DHA

Gene-ID	Symbol	Description
PF3D7_1148500	NA	non-coding RNA
PF3D7_1203800	NA	non-coding RNA
PF3D7_0919600	WBP1	WBP1 DDP glycosyltransferase
PF3D7_1310000	OSCP	ATP synthase subunit O, mitochondrial, putative
PF3D7_1478900	NA	non-coding RNA
PF3D7_0931200	Sel2	selenoprotein
PF3D7_1209800	ATP11	ATP synthase mitoc. F1 complex assembly factor 1
PF3D7_0107700	NA	OST3/OST6, DDP glycosyltransferase, putative
PF3D7_1313200	MTFMT	methionyl-tRNA formyltransferase, putative
PF3D7_1200700	ACS7	acyl-CoA synthetase-7
PF3D7_0202200	PTP1	EMP1-trafficking protein
PF3D7_1370300	MAHRP1	membrane associated histidine-rich protein 1
PF3D7_1001200	ACBP2	acyl-CoA binding protein, isoform 2, ACBP2
PF3D7_0501400	FIRA	interspersed repeat antigen
PF3D7_0424700	FIKK4.2	serine/threonine protein kinase, FIKK family
PF3D7_0731400	FIKK7.2	serine/threonine protein kinase, FIKK family
PF3D7_1001100	ACBP1	acyl-CoA binding protein, isoform 1, ACBP1
PF3D7_0215300	ACS8	acyl-CoA synthetase
PF3D7_0501100	HSP40	heat shock protein 40, type II
PF3D7_1477800	ACBP	acyl-CoA binding protein
PF3D7_1105600	PTEX88	translocon component PTEX88
PF3D7_0919300	TrxL1	thioredoxin-like protein 1, putative
PF3D7_0831700	HSP70-X	heat shock protein 70
PF3D7_1479000	ACS1a	acyl-CoA synthetase
PF3D7_0902500	FIKK9.6	serine/threonine protein kinase, FIKK family
PF3D7_0104300	UBP1	ubiquitin carboxyl-terminal hydrolase 1, putative
PF3D7_0708500	HSP86	heat shock protein 86 family protein
PF3D7_1102300	NA	Plasmodium exported protein, unknown function
PF3D7_1341100	NA	U6 spliceosomal RNA
PF3D7_0308400	NA	U4 spliceosomal RNA
PF3D7_0922900	FabG	3-oxoacyl-[acyl-carrier-protein] reductase
PF3D7_0816500	HSP20	small heat shock protein HSP20, putative
PF3D7_0731600	ACS5	acyl-CoA synthetase
PF3D7_0511200	SCD	stearoyl-CoA desaturase
PF3D7_1454100	NA	tRNA intron endonuclease, putative
PF3D7_1248000	NA	tRNA-splicing endonuclease, putative
PF3D7_0935100	KSH1	protein kish, putative
PF3D7_0901800	NA	Plasmodium exported protein
PF3D7_0221200	NA	Plasmodium exported protein (hyp15)
PF3D7_1467300	DXR	1-deoxy-D-xylulose 5-phosphate reductoisomerase

BTZ

Gene	Symbol	Description
PF3D7_1148500	NA	non-coding RNA
PF3D7_1215000	Trx-Px2	thioredoxin peroxidase 2
PF3D7_1209200	LSM7	U6 snRNA-associated Sm-like protein LSm7
PF3D7_1344500	USB1	U6 snRNA phosphodiesterase, putative
PF3D7_0922900	FabG	3-oxoacyl-[acyl-carrier-protein] reductase
PF3D7_1209800	ATP11	ATP synthase mitoc. F1 complex assembly factor 1
PF3D7_0802200	1-CysPxn	1-cys peroxiredoxin
PF3D7_1200700	ACS7	acyl-CoA synthetase
PF3D7_0202200	PTP1	EMP1-trafficking protein
PF3D7_0501400	FIRA	interspersed repeat antigen
PF3D7_0731600	ACS5	acyl-CoA synthetase
PF3D7_0424700	FIKK4.2	serine/threonine protein kinase, FIKK family
PF3D7_1337200	DXS	1-deoxy-D-xylulose 5-phosphate synthase
PF3D7_1370300	MAHRP1	membrane associated histidine-rich protein 1
PF3D7_1479000	ACS1a	acyl-CoA synthetase
PF3D7_0308400	NA	U4 spliceosomal RNA
PF3D7_0907700	PA28	proteasome activator 28
PF3D7_0831700	HSP70-X	heat shock protein 70
PF3D7_0902500	FIKK9.6	serine/threonine protein kinase, FIKK family
PF3D7_1001100	ACBP1	acyl-CoA binding protein, isoform 1, ACBP1
PF3D7_0215300	ACS8	acyl-CoA synthetase
PF3D7_0731400	FIKK7.2	serine/threonine protein kinase, FIKK family, pseudogene
PF3D7_0104300	UBP1	ubiquitin carboxyl-terminal hydrolase 1, putative
PF3D7_1001200	ACBP2	acyl-CoA binding protein, isoform 2, ACBP2
PF3D7_1454100	NA	tRNA intron endonuclease, putative
PF3D7_1248000	NA	tRNA-splicing endonuclease, putative
PF3D7_0935100	KSH1	protein kish, putative
PF3D7_0901800	NA	Plasmodium exported protein, unknown function
PF3D7_0221200	NA	Plasmodium exported protein (hyp15), unknown function
PF3D7_0919300	TrxL1	thioredoxin-like protein 1, putative
PF3D7_1105100	H2B	histone H2B
PF3D7_1203900	UBC	ubiquitin-conjugating enzyme E2
PF3D7_0314100	NA	vesicle transport v-SNARE protein, putative
PF3D7_1243600	NA	translation initiation factor SUI1, putative
PF3D7_1030000	SPT4	transcription elongation factor SPT4, putative
PF3D7_0617900	H3.3	histone H3 variant
PF3D7_0501100	HSP40	heat shock protein 40, type II
PF3D7_1116800	HSP101	heat shock protein 101
PF3D7_1436300	PTEX150	translocon component PTEX150
PF3D7_1477800	ACBP	acyl-CoA binding protein
PF3D7_0104200	NA	StAR-related lipid transfer protein
PF3D7_1203800	NA	non-coding RNA
PF3D7_1345100	TRX2	thioredoxin 2
PF3D7_1121600	EXP1	exported protein 1
PF3D7_0816500	HSP20	small heat shock protein HSP20, putative
PF3D7_1471100	EXP2	exported protein 2

- Up in both (NF54 and *pB*104)
- Up only in NF54
- Up only in *pB*104
- Up *pB*104 down NF54
- Down in both (NF54 and *pB*104)
- Down only in *pB*104
- Down only in NF54
- Down *pB*104 up NF54

Figure S6. DHA and BTZ differential expression genes from RNAseq analysis (see Method) comparison between *piggybac* sensitive mutant, *pB*104, and NF54 wild-type. The entire list of differentially expressed genes is provided in DataSet S4 and S5.

Figure S7

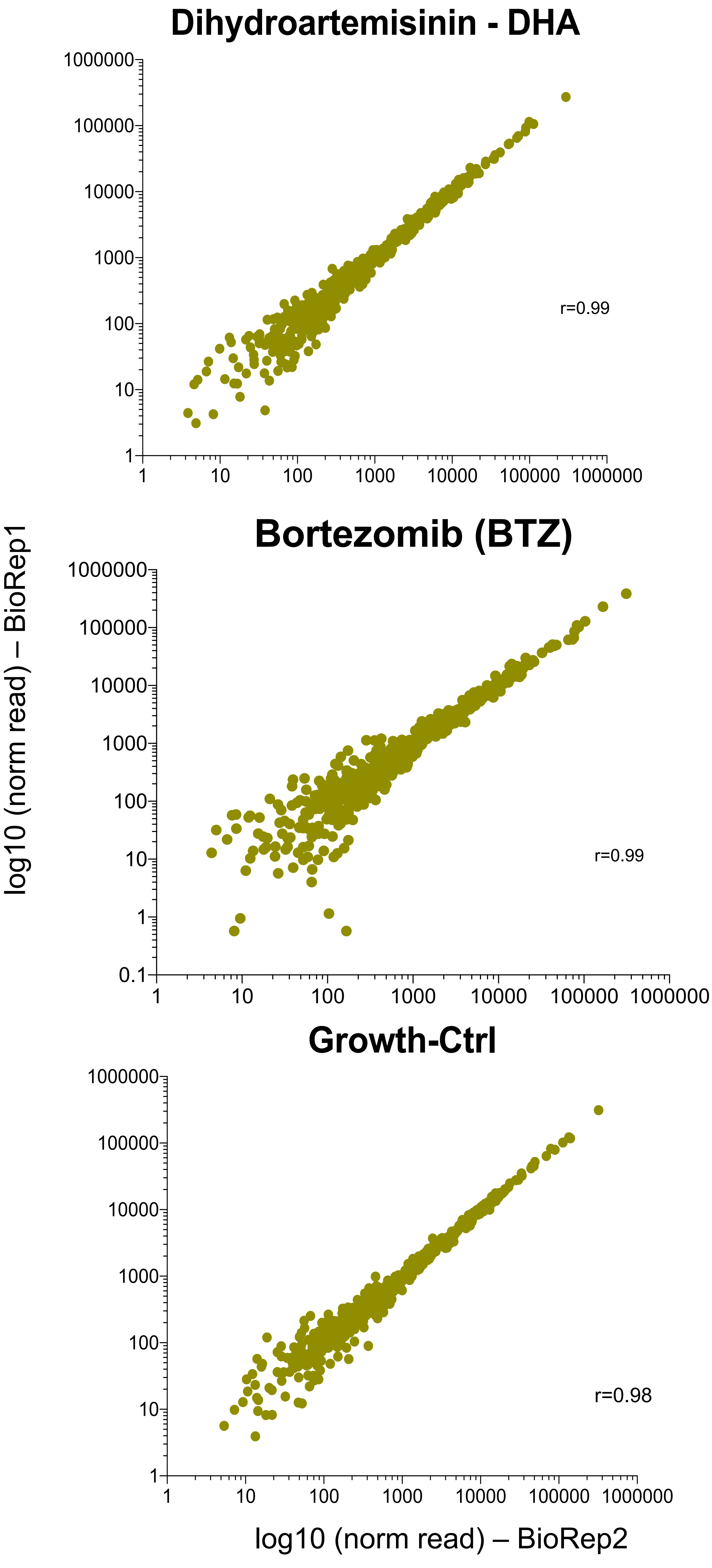

Figure S7. Person correlation between biological replicates of each drug (DHA and BTZ) screens and no-drug growth-control

Figure S8

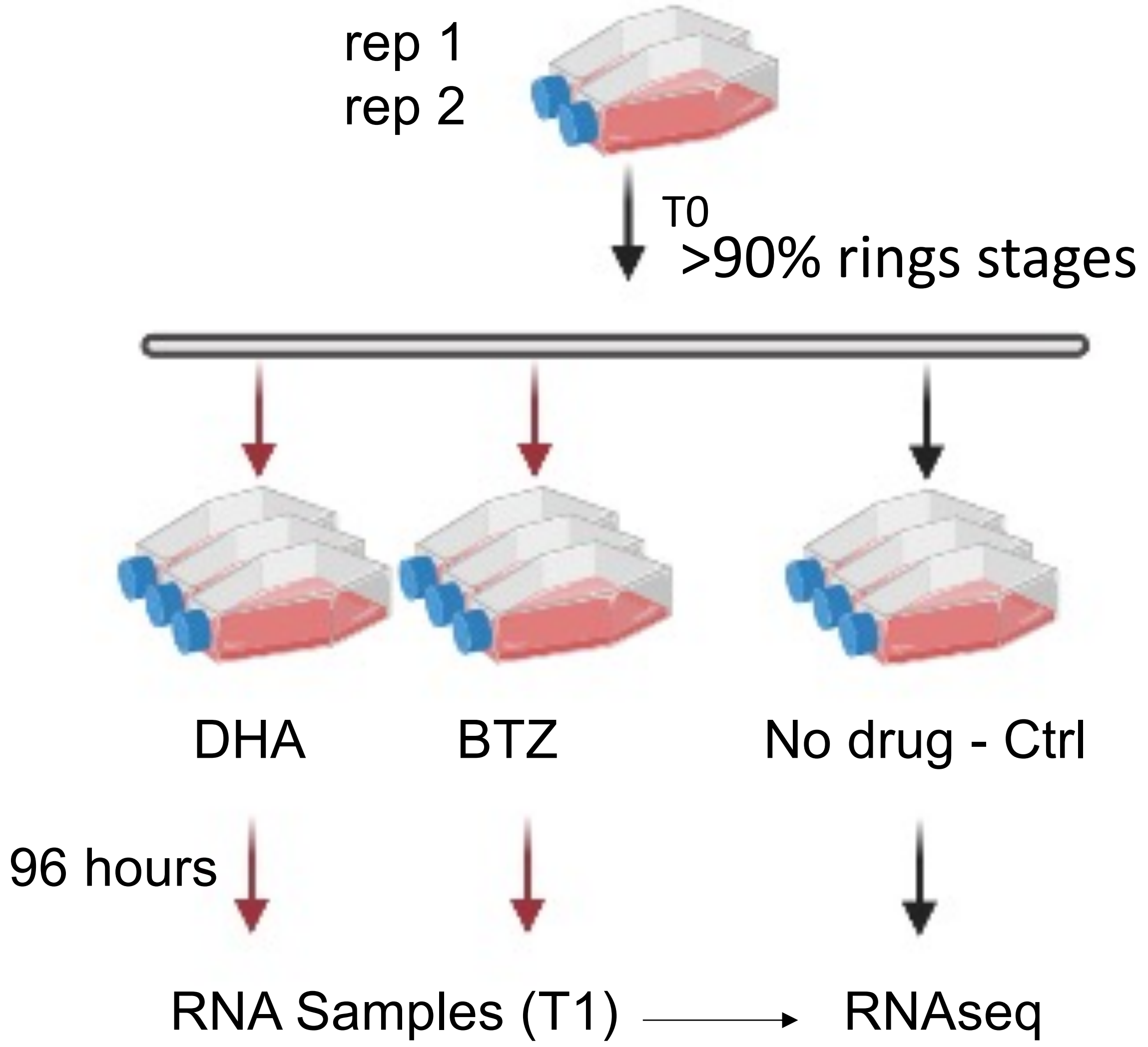

Figure S8. The experiment approach of RNAseq. Two biological replicates were performed. Cultures of wildtype NF54 and sensitive mutant *pB*104 were synchronized to rings, the timepoint zero (T0) were harvested, then split equally into experimental flasks (DHA and BTZ) and Control flasks (no-drug growth control). All parasites were grown at the normal culture condition. Experimental flasks of each parasite-line were then exposed to 96 hours of continuous drug-pressure at sublethal dose (4 nM) of DHA and BTZ (40 nM). Control-flasks were cultured continuously in parallel at 37°C without drug. After the 96 hours the RNA was harvested simultaneously from all conditions for RNAseq. Parasitemia was verified by Giemsa smear at every day. RNAseq was performed on an Illumina NextSeq V2.5 mid-output 300-cycle, Trueseq reagent kit (see Methods).

Normalized reads (DEseq2) correlation

Figure S9

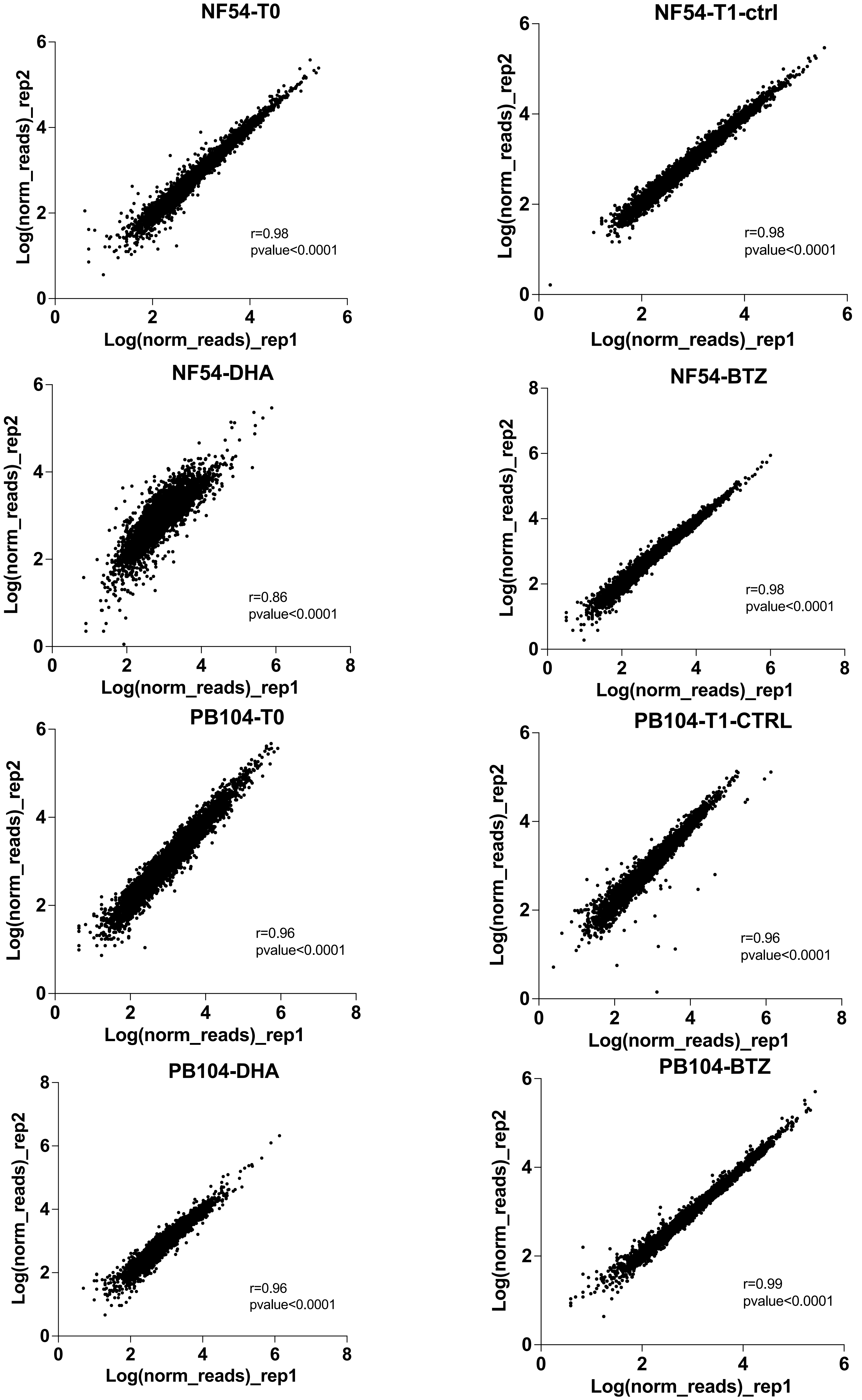

Figure S9. Pearson correlation between biological replicates of RNAseq data of NF54 no-drug controls in two time points (NF54_T0, NF54_T1_control), and treated with DHA (NF54_DHA) and BTZ (NF54_BTZ), *pB*104 no-drug controls in two time points (*pB*104_T0, *pB*104_T1_control), and treated with DHA (*pB*104_DHA) and BTZ (*pB*104 _BTZ). The correlation analysis was performed to DEseq2 normalized reads.
