# Supplementary material for: Chemogenomic Profiling of a Plasmodium falciparum Transposon Mutant Library Reveals Shared Effects of Dihydroartemisinin and Bortezomib on Lipid Metabolism and Exported Proteins
Source: Microbiol Spectr. 2023 Apr 17;11(3):e05014-22. doi: 10.1128/spectrum.05014-22 (PMC10269874; doi:10.1128/spectrum.05014-22)
Supplement: Supplemental file 2 — Guide to supplemental data sets and tables. Download spectrum.05014-22-s0002.pdf, PDF file, 0.07 MB [file spectrum.05014-22-s0002.pdf]

Guide to Supplementary Data

DataSet S1: QIseq data info

Tab1: Raw QIseq data sets generated for this study were deposited to the European Nucleotide Archive under study accession code ERP114305

Tab 2: Original reads mapping generated by QIseq

DataSet S2: Half k screens DEseq outputs

Tab1: DHA_treated

Tab2: BTZ_treated

Tab 3: No-Drug_Ctrl_treated

DataSet S3: MPMP enrichment

Tab1: Tolerants (DHA and BTZ)

Tab2: Sensitives (DHA and BTZ)

DataSet S4: NF54 RNAseq analysis, DEseq2 out
Tab 1: No-Drug_Ctrl_treated

Tab 2: DHA_treated

Tab 3: BTZ_treated

DataSet S5: pB104 RNAseq analysis, DEseq2 out
Tab 1: No-Drug_Ctrl_treated

Tab 2: DHA_treated

Tab 3: BTZ_treated

DataSet S6: GO enrichment
Tab 1: merged DHA_BTZ

Tab 2: DHA categories

Tab 3: BTZ categories

Table S1: GO enrichment of half K library; data referred to Figure 1B

Table S2: GO enrichment DHA and BTZ phenotype (sensitive and tolerant); data referred to Figure 2B
